# Sexual dimorphism in the molecular mechanisms of insulin resistance during a critical developmental window in *Wistar* rats

**DOI:** 10.1186/s12964-022-00965-6

**Published:** 2022-10-12

**Authors:** Rosa Isela Ortiz-Huidobro, Carlos Larqué, Myrian Velasco, Juan Pablo Chávez-Maldonado, Jean Sabido, Yuriko Itzel Sanchez-Zamora, Marcia Hiriart

**Affiliations:** 1 Neurosciences Division, Department of Cognitive Neuroscience, Instituto de Fisiología Celular, Universidad Nacional Autónoma de México, Mexico City, Mexico; 2 Department of Embryology, and Genetics, Facultad de Medicina, Universidad Nacional Autónoma de México, Mexico City, Mexico

**Keywords:** Insulin resistance, Sexual dimorphism, Insulin signaling pathway, Critical developmental window, Glucose homeostasis, S6K1 kinase, IRS1, GLUT4, Adipose tissue, Skeletal muscle

## Abstract

**Background:**

Insulin resistance (IR) is a condition in which the response of organs to insulin is impaired. IR is an early marker of metabolic dysfunction. However, IR also appears in physiological contexts during critical developmental windows. The molecular mechanisms of physiological IR are largely unknown in both sexes. Sexual dimorphism in insulin sensitivity is observed since early stages of development. We propose that during periods of accelerated growth, such as around weaning, at postnatal day 20 (p20) in rats, the kinase S6K1 is overactivated and induces impairment of insulin signaling in its target organs. This work aimed to characterize IR at p20, determine its underlying mechanisms, and identify whether sexual dimorphism in physiological IR occurs during this stage.

**Methods:**

We determined systemic insulin sensitivity through insulin tolerance tests, glucose tolerance tests, and blood glucose and insulin levels under fasting and fed conditions at p20 and adult male and female *Wistar* rats. Furthermore, we quantified levels of S6K1 phosphorylated at threonine 389 (T389) (active form) and its target IRS1 phosphorylated at serine 1101 (S1101) (inhibited form). In addition, we assessed insulin signal transduction by measuring levels of Akt phosphorylated at serine 473 (S473) (active form) in white adipose tissue and skeletal muscle through western blot. Finally, we determined the presence and function of GLUT4 in the plasma membrane by measuring the glucose uptake of adipocytes. Results were compared using two-way ANOVA (With age and sex as factors) and one-way ANOVA with post hoc Tukey’s tests or t-student test in each corresponding case. Statistical significance was considered for *P* values < 0.05.

**Results:**

We found that both male and female p20 rats have elevated levels of glucose and insulin, low systemic insulin sensitivity, and glucose intolerance. We identified sex- and tissue-related differences in the activation of insulin signaling proteins in p20 rats compared to adult rats.

**Conclusions:**

Male and female p20 rats present physiological insulin resistance with differences in the protein activation of insulin signaling. This suggests that S6K1 overactivation and the resulting IRS1 inhibition by phosphorylation at S1101 may modulate to insulin sensitivity in a sex- and tissue-specific manner.

**Video Abstract**

**Supplementary Information:**

The online version contains supplementary material available at 10.1186/s12964-022-00965-6.

## Plain English summary

Insulin regulates the synthesis of carbohydrates, lipids and proteins differently between males, and females. One of its primary functions is maintaining adequate blood glucose levels favoring glucose entry in muscle and adipose tissue after food consumption. Insulin resistance (IR) is a condition in which the response of organs to insulin is impaired. IR is frequently associated with metabolic dysfunction such as inflammation, obesity, or type 2 diabetes. However, physiological IR develops in healthy individuals during periods of rapid growth, pregnancy, or aging by mechanisms not fully understood. We studied the postnatal development, specifically around weaning at postnatal day 20 (p20) of Wistar rats. In previous works, we identified insulin resistance during this period in male rats. This work aimed to characterize IR at p20, determine its underlying mechanisms, and identify whether sexual dimorphism in physiological IR occurs during this stage. We found that p20 rats of both sexes have elevated blood glucose and insulin levels, low systemic insulin sensitivity, and glucose intolerance. We identified differences in insulin-regulated protein activation (S6K1, IRS1, Akt, and GLUT4) between sexes in different tissues and adipose tissue depots. Studying these mechanisms and their differences between males and females is essential to understanding insulin actions and their relationship with the possible development of metabolic diseases in both sexes.

**Supplementary Information:**

The online version contains supplementary material available at 10.1186/s12964-022-00965-6.

## Background

Insulin is an anabolic hormone that regulates the metabolism of carbohydrates, lipids, and proteins. One of its primary functions is maintaining glucose homeostasis by favoring glucose internalization in skeletal muscle and adipose tissue through the glucotransporter 4 (GLUT4). In the liver, insulin promotes glycogen synthesis, inhibits gluconeogenesis, and regulates the synthesis and release of lipids. In muscle, insulin promotes glycogen synthesis, while in adipose tissue, this hormone increases lipogenesis and inhibits lipolysis [1].

The canonical pathway of phosphatidylinositol 3-kinase/protein kinase B (PI3K/Akt) mainly regulates the metabolic actions of insulin. In the PI3K/Akt signaling pathway, the activated insulin receptor phosphorylates the insulin receptor substrate (IRS) at tyrosine residues. Subsequently, IRS activates PI3K, which produces phosphatidylinositol 3,4,5-trisphosphate (PIP3). In the membrane, PIP3 constitutes a binding site to phosphoinositide-dependent kinase-1 (PDK1). Finally, PDK1 and the mammalian target of rapamycin complex 2 (mTORC2) phosphorylate and activate the serine/threonine kinase Akt which is a critical node of insulin signaling because it modulates the activity of a wide variety of substrates [1].

Insulin resistance (IR) is a condition in which the response of organs to insulin is impaired. There are high blood glucose and insulin levels in IR during fasting and postprandial periods. Increased blood glucose levels are related to increased hepatic glucose production and impaired glucose internalization by skeletal muscle and adipose tissue [1]. The subsequent rise in plasma insulin levels may result from a sustained beta-cell activity stimulated by high glucose concentrations or their response to meta-inflammation and lipotoxicity [2]. In addition, insulin-mediated inhibition of lipolysis is lost in adipose tissue with IR, which results in an increased release of free fatty acids toward the bloodstream [1].

The molecular mechanisms described in the IR are diverse. They are generally related to an excess of nutrients (e.g., branched-chain amino acids), inflammation, lipotoxicity, mitochondrial dysfunction, and endoplasmic reticulum stress [1, 3, 4]. A typical event in these mechanisms is the desensitization of insulin signaling by the action of serine and threonine kinases [5].

The mammalian target of rapamycin complex 1 (mTORC1) and its downstream target protein, ribosomal S6 kinase 1 (S6K1), regulate insulin-induced protein synthesis and have emerged as critical signaling components in obesity-induced IR due to homologous desensitization of the PI3K/Akt pathway [6]. Excess nutrients and hyperinsulinemia induce mTORC1 and S6K1 overactivation, which further downregulate IRS1 transcription and promote IRS1 degradation. S6K1 kinase phosphorylates IRS1 at serine 1101 (S1101), inhibiting its function and impairing insulin signaling [7]. It has been observed that S6K1 overactivation may disrupt PI3K signaling and inhibits glucose internalization in L6 myocytes and 3T3-L1 adipocytes [8]. On the other hand, S6K1 deficient mice fed with a high-fat diet maintain normal glucose levels during fasting. They show higher insulin sensitivity than their controls, protecting them against obesity-induced IR [9].

It is well accepted that multiple factors such as growth hormone (GH) may impair insulin signaling. Experimental work suggests that GH could be involved in developing physiological IR during puberty [10]. GH could inhibit IRS/PI3K/Akt pathway via JAK2/STAT5 pathway [11, 12]. Nevertheless, it is unknown whether these mechanisms could be involved in the impairment of insulin sensitivity during the early stages of development.

Sex is a factor involved in insulin sensitivity [13–15]. Since the early stages of development, sexual dimorphism in metabolism is observed. It may be influenced by differential gene expression, epigenetic regulation, metabolic programming during gestation, and sex hormones which may modulate insulin secretion, insulin sensitivity, and glucose homeostasis [16]. Several works using murine models of diet-induced IR have observed that males develop more severe metabolic dysfunction than females [13–15].

Moreover, sex-related differences in insulin signaling have been observed in obesity and metabolic syndrome animal models [17], which suggests that mechanisms of energetic homeostasis show sexual dimorphism in response to high fat- or high carbohydrates-diets [13, 14].

IR is an early marker of metabolic dysfunction associated with inflammation and obesity. It is a central component of metabolic syndrome and a common feature in the history of patients who develop type 2 diabetes mellitus (T2DM) [2, 5, 18]. However, IR also develops in physiologic contexts during critical developmental windows, such as rapid growth periods, pregnancy, or aging, as a response to variations in energy demand [19–23].

We are interested in the physiological changes during early postnatal development, specifically around weaning in *Wistar* rats (that under laboratory conditions occurs at postnatal day 20 (p20)). During this critical developmental window, which lasts approximately eight days, previous works by Hiriart and collaborators identified changes related to the functional maturation of pancreatic beta cells [22, 24–26] and observed systemic IR in male p20 rats [22].

The molecular mechanisms underlying physiological IR may explain some risk factors for developing metabolic diseases. According to previous works using different models of obesity-induced IR, we hypothesized that hyperactivity of both mTORC1 and S6K1 may be induced by greater demand for protein synthesis observed during periods of accelerated growth [20], and may be involved in the physiological desensitization of insulin signaling in organs with high metabolic rate.

This work aimed to characterize IR at p20, determine its underlying mechanisms, and identify whether sexual dimorphism in physiological IR occurs during this stage. We found that both male and female p20 rats show elevated blood glucose and insulin levels, low systemic insulin sensitivity, and glucose intolerance. However, we identified differences in S6K1, IRS1, Akt phosphorylation, and the presence of GLUT4 in the membrane between sexes, tissues, and in different white adipose tissue (WAT) depots.

## Methods

Systemic insulin sensitivity and phosphorylation levels of insulin signaling proteins in WAT and skeletal muscle were assessed under fasting and fed conditions in p20 and adult (control group) male and female *Wistar* rats.

### Animals

Male and female at postnatal day 20 (p20) (45–50 g of body weight) and young adult *Wistar* rats (250–280 g of body weight, approximately 8 weeks of age) were obtained from the local facility of the IFC, UNAM, Mexico City. Rats were housed in 12 h light/dark cycle, at 20–23 °C and 40% of relative humidity.

All animals were fed with a standard rat chow diet (Lab Diet 5001) composed by 28.5% protein, 13.5% fat, and 58% carbohydrates. Ad libitum tap water was provided. Rats were randomly separated into fasting and fed groups. Adult rats were fasted for 12 h prior to every experiment (9:00 a.m.), because they had stable glucose levels after this fasting period (around 70 mg /dL); while we only fasted p20 rats for 4 h, were we observed that they reach basal glucose levels. Food access stayed constant before trials to assess the postprandial condition.

Animals were anesthetized with an intraperitoneal injection of sodium pentobarbital (40 mg/kg) to obtain tissue samples. Blood samples were obtained from the inferior cava vein in heparinized tubes. Peripancreatic (pWAT) and gonadal (gWAT) white adipose tissue samples, and gastrocnemius muscles were dissected. Body weight and weight of each tissue were reported. Animals were euthanized with a sodium pentobarbital overdose (100–150 mg/kg) followed by cervical dislocation.

### ITT (insulin tolerance test) and IPGTT (intraperitoneal glucose tolerance test)

We performed the ITT or IPGTT (on 4 or 12 h fasted-rats, p20 or adult rats, respectively) in adult rats one week prior to tissue dissection. IP insulin (0.2 IU/Kg of body weight) or glucose (2 g glucose/Kg of body weight) injection was administered to each rat. The animals were handled by the same person. Peripheral blood samples were collected from the tail vein immediately before glucose or insulin administration (time 0) and after 15, 30, 60, 90 and 120 min. Glucose levels were determined with a glucometer (Accu-Check, Hoffman La Roche Basel Switzerland).

### Hormones and cytokines in blood circulation

We determined plasma insulin levels using the Ultrasensitive rat insulin ELISA system according to manufacturer’s instructions (10-1137-10; Mercodia Uppsala, Sweden). The plasma levels of TNFα, IL-1β, and growth hormone (GH), were determined by ELISA (Preprotech) according to the manufacturer’s instructions. Optical density (OD) was measured after 15 min using an ELISA reader (Multiskan FC Microplate Photometer, Thermo Scientific) at 450 nm to insulin, and 405 nm to TNFα, IL-1β, and GH.

### Insulin-stimulated glucose uptake assay

Insulin-stimulated glucose uptake was determined using a Glucose uptake cell-based assay kit according to manufacturer’s instructions (600,470; Cayman Chemical, USA). Briefly, duplicates of gonadal adipose tissue (50 mg) were incubated 30 min at 37 °C in Hank’s solution with 2-N(-7-nitrobenz-2-oxa-1,3-diazol-4-yl)-2-deoxyglucose (2-NBDG glucose) 100 mg/mL (control condition), and Hank’s solution with 2-NBDG glucose 100 mg/mL and insulin 100 nM (stimulating condition). Glucose uptake was measured using a Flex Station 3 Multi-mode Microplate Reader (Molecular Devices). Results were expressed as a percentage of increase compared to control without insulin.

### Protein extraction and Western blot

Pooled samples (n = 15 for p20 rats, and n = 5 for adult rats) of pWAT, gWAT, or gastrocnemius muscle protein extracts were obtained by homogenization in RIPA lysis buffer. Total protein was quantified by Bradford’s method. The protein extracts were separated by SDS-PAGE in 8% polyacrylamide gels and transferred to a PVDF membrane using an accelerated semi-dry blotting transfer system (Trans-blot turbo, Bio-Rad Laboratories, Hercules CA, USA). The membranes were blocked with non-fat dry milk 5% in TBS-tween 1% for 1 h at room temperature and incubated overnight at 4 °C with rabbit polyclonal anti-Akt (CST#9272) or rabbit polyclonal anti-p-Akt(S473) (CST#9271) diluted 1:800; rabbit polyclonal anti-P70S6K1 (CST#9202) or rabbit polyclonal anti-p-P70S6K1(T389) (CST#9205) diluted 1:800; rabbit polyclonal anti-IRS1 (CST#2382) or rabbit polyclonal anti-p-IRS1(S1101) (CST#2385) diluted 1:200; mouse monoclonal anti-GLUT4 (SCB #sc53566) diluted 1:500 in blocking solution. Goat polyclonal anti-β-actin (SCB #sc1616) diluted 1:3500 and mouse monoclonal anti-Na^+^/K^+^-ATPase α (SCB #sc48345) diluted 1:5000 in blocking solution were used for the detection of load control proteins. After incubation with secondary antibodies, proteins were visualized using a chemiluminescent HRP substrate method with Immobilon Western Chemiluminescent HPR substrate (#WBKLSO100, Merck Millipore, Mass, USA). Densitometric analysis was performed using ImageJ software (NIH 1.44) and represents an average of the intensity of bands of three or two independent experiments. See supplementary images from western blots in Additional file 1.

### Adipocytes culture and labeling of membrane proteins

Isolated adipocytes from p20 and adult rats were obtained following a previously described procedure [27]. Briefly, after dissecting tissues, gWAT was fragmented and digested with collagenase P (0.5 mg/mL to p20 tissue or 1 mg/mL to adult tissue) in Hank’s balanced salt solution, during 7 min (p20 tissue samples) or 15 min (adult tissue samples), at 37 °C in a shaking bath. Once a homogeneous mixture was obtained, cold Hank's solution was added, and samples were centrifuged at 800 rpm for 5 min. Mature adipocytes were collected from the solution surface, and cell count was performed by using trypan blue dye staining in a Neubauer chamber. Before the experiments, adipocytes were incubated in DMEM F12 medium containing 1% FBS (fetal bovine serum) and 10 mM glucose overnight.

Adipocytes from gonadal depot were incubated with insulin 1 nM or DMSO 0.1% in DMEM medium for 30 min. Plasma membrane proteins were labeled with sulfo-NHS-SS-biotin using Pierce cell surface protein isolation kit according to manufacturer’s instructions (89,881; Thermo Scientific, USA). Briefly, adipocytes were incubated with an EZ-Link sulfo-NHS-SS-biotin solution for 30 min at 4 °C, and a quenching solution was added to stop the reaction. Subsequently, cells were lysed with moderate activity detergents for 30 min at 4 °C. Cell lysates were separated by centrifugation at 13,000 rpm, 5 min, 4 °C. Labeled proteins were separated by incubating samples in a column with avidine agarose beads during 1 h at room temperature. Labeled proteins (60 μg protein) were separated by SDS-PAGE electrophoresis in 8% polyacrylamide gels and subjected to analysis by Western blot as described above. Three independent experiments were performed.

### Statistical analysis

All data are reported as mean ± S.E.M.; “n” denotes the number of animals analyzed. For statistical analysis, two-way ANOVA (With age and sex as factors) and one-way ANOVA with post hoc Tukey’s tests, or t-student test were performed (GraphPad Prism version 9.3.1 (471)). Statistical significance was considered for *P* values < 0.05.

## Results

### Male and female Wistar rats at p20 exhibit systemic physiological IR in a similar way

We first determined systemic insulin sensitivity under fasting and fed conditions in both sexes’ p20 and adult rats.

**Fig. 1:**
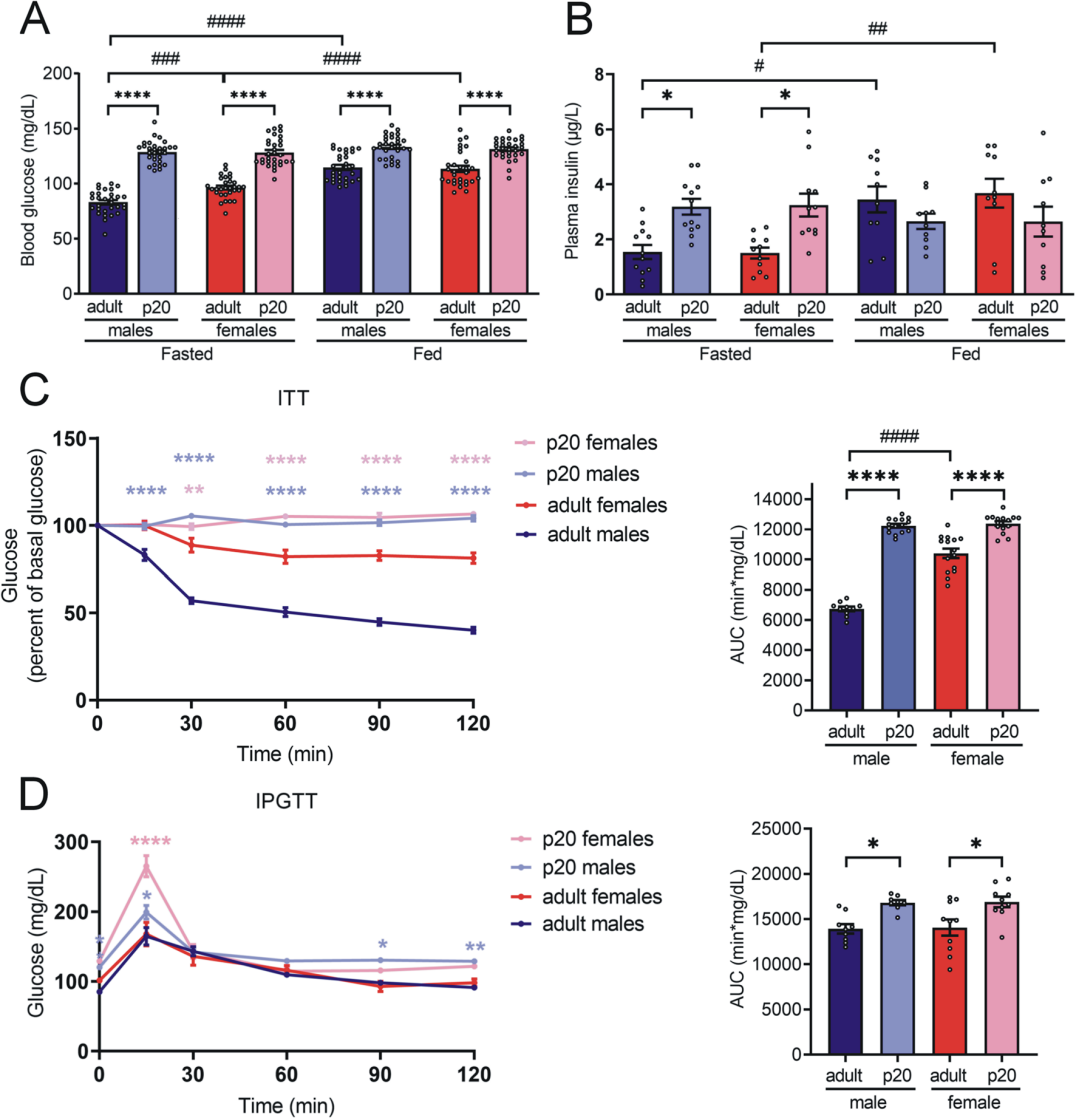
Systemic insulin sensitivity and glucose tolerance.** a** Blood glucose, n = 30 rats per group and **b** plasma insulin, n = 10 rats per group. The bars represent the mean ± S.E.M. *P < 0.05, ****P < 0.0001 compared to their control group by two-way ANOVA with post hoc Tukey's test; ^#^*P* < 0.05, ^##^*P* < 0.005, ^###^*P* < 0.0005, ^####^*P* < 0.0001 compared between sex and fasting vs fed state by two-way ANOVA with post hoc Tukey’s test; **c** Insulin sensitivity test (ITT), 0.2 IU/kg of body weight of intraperitoneal insulin was administered. Data expressed as mean ± S.E.M., n = 15 rats per group. **P < 0.005, ****P < 0.0001 compared to their control and in ITT area under the curve (AUC), the bars represent the mean ± S.E.M. ****P < 0.0001 compared to their control group and ^####^*P* < 0.0001 compared between sex by one-way ANOVA with post hoc Tukey’s test; **d** IP glucose tolerance test (IPGTT), 2 g/kg of body weight of intraperitoneal glucose was administered. Data expressed as mean ± S.E.M., n = 10 rats per group. *P < 0.05, **P = 0.007, ****P < 0.0001 compared to their control and in IPGTT area under the curve (AUC), the bars represent the mean ± S.E.M. *P < 0.05 compared to their control group and compared between sex by one-way ANOVA with post hoc Tukey’s test. Created with GraphPad Prism V 9.3.1 (471)

The p20 rats showed higher fasting blood glucose and plasma insulin concentrations than adult rats (Fig. 1a, b). As expected, there were a rise in blood glucose and plasma insulin levels in fed adult animals. However, glucose and insulin concentrations remained constant in fed p20 rats of both sexes. We did not observe significant differences in glucose and insulin levels between sexes at p20.

We performed insulin tolerance tests (ITT) to determine systemic insulin sensitivity and observed that both male and female p20 rats showed higher concentrations of blood glucose in a sustained manner after administration of intraperitoneal insulin compared to adults (Fig. 1c). Furthermore, we did not observe any difference in insulin sensitivity between p20 males and females, as seen in the area under the curve (AUC) graph. In adult rats, we observed a lower insulin sensitivity in females than in males.

We also explored beta-cell insulin secretion through the intraperitoneal glucose tolerance test (IPGTT, Fig. 1d). At the end of the test, we observed that p20 males showed higher blood glucose levels than adult males. It is worth noting that, p20 females only showed higher glucose levels at 15 min after glucose administration compared to adult females. However, the AUC graph depicts that p20 rats of both sexes show lower glucose tolerance than adult rats.

### Physiological IR in p20 rats is not associated with obesity and inflammation

**Fig. 2:**
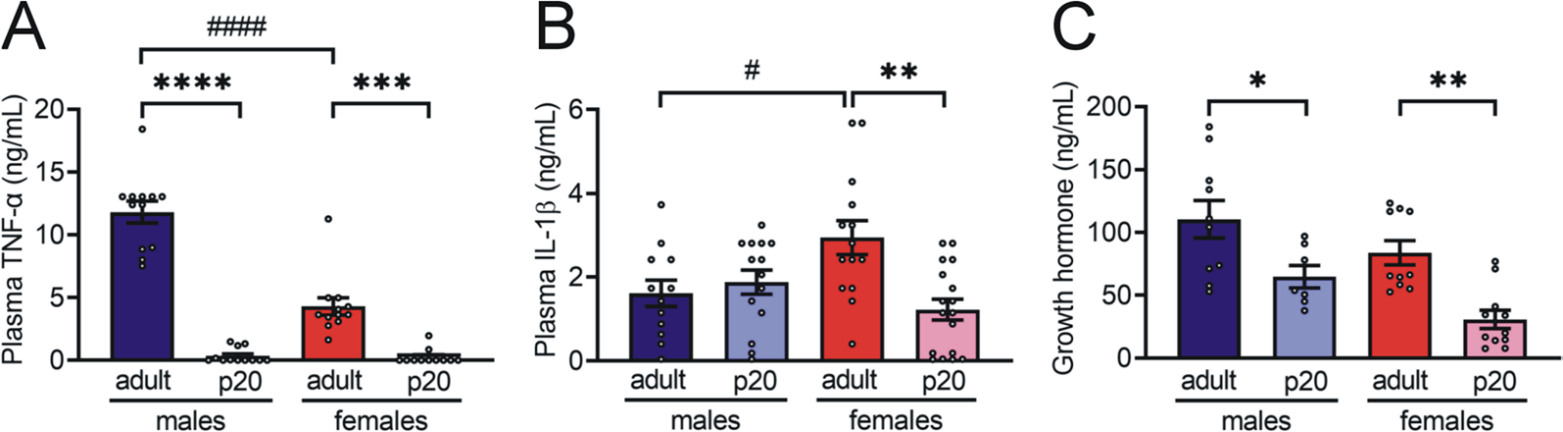
Plasma hormones and cytokines associated with IR mechanisms. **a** Plasma TNF-α, **b** plasma IL-1β, and **c** plasma GH. The bars represent the mean ± S.E.M., n = 10–12 rats per group. *P < 0.05, **P < 0.005, ***P = 0.0001, ****P < 0.0001 compared to their control group by one-way ANOVA with post hoc Tukey's test; ^#^*P* < 0.05, ^####^*P* < 0.0001 compared between sex by one-way ANOVA with post hoc Tukey’s test. Created with GraphPad Prism V 9.3.1 (471)

In obesity-induced IR, adipose tissue accumulation and its dysfunction result in the secretion of proinflammatory cytokines that impair insulin signaling [28]. To explore if physiological IR is associated with these mechanisms, we determine the weight of two body fat depots, peripancreatic white adipose tissue (pWAT) and gonadal white adipose tissue (gWAT), and the gastrocnemius muscle. We did not find significant differences in these parameters between animals of both sexes (Table 1). In addition, we found lower plasma levels of the proinflammatory cytokines such as tumor necrosis factor α (TNFα) and interleukin-1β (IL-1β) in p20 rats compared to adults (Fig. 2a, b). In addition, female adult rats showed lower levels of TNFα and higher levels of IL-1β compared to male adult animals. On the other hand, we measured growth hormone (GH) levels due to its known inhibitory effects on insulin metabolic actions [11, 12]. We found lower GH levels in p20 rats than in adult rats (Fig. 2c).

**Table 1:** Body and tissues weight

	Adult rats		p20 rats	
	Male	Female	Male	Female
Body weight (g)	289.7 ± 4.9	290.3 ± 1.1^ns^	50.0 ± 0.1	49.7 ± 0.2^ns^
Peripancreatic fat (g)	0.874 ± 0.03	0.804 ± 0.02^ns^	0.047 ± 0.007	0.039 ± 0.005^ns^
Gonadal fat (g)	3.22 ± 0.07	3.43 ± 0.06^ns^	0.07 ± 0.008	0.06 ± 0.005^ns^
Gastrocnemius muscle (g)	1.22 ± 0.06	1.22 ± 0.05^ns^	0.16 ± 0.01	0.14 ± 0.02^ns^

Data expressed as mean ± S.E.M., n = 30 for body weight and n = 5 for tissue weight, groups were compared between sex by unpaired t-student test. Statistical significance was considered for *P* values < 0.05. ns = not significantly different with respect to weight in males

### Proteins of the insulin signaling pathway are activated in sex-, and tissue-specific manner in p20 rats

We identified systemic IR is similar in male and female p20 rats. However, we further explored the molecular mechanisms contributing to insulin signaling desensitization in its target organs during this developmental stage in both sexes. We explored insulin sensitivity assessing the PI3K/Akt signaling pathway in pWAT and gWAT due to their proximity to the pancreas and sexual glands, respectively [29]. On the other hand, we also explored the PI3K/Akt signaling pathway in gastrocnemius muscle, mainly composed of glycolytic muscle fibers with high glucose utilization [30].

To further analyze the inhibition of the insulin signaling pathway mediated by S6K1 activity, we determined the levels of S6K1 phosphorylated at threonine 389 (T389) (active form) and its target IRS1 phosphorylated at serine 1101 (S1101) (inhibited form). In addition, we assessed insulin signal transduction by measuring levels of Akt phosphorylated at serine 473 (S473). Finally, we determined GLUT4 membrane expression levels and function in gonadal adipocytes.

**Fig. 3:**
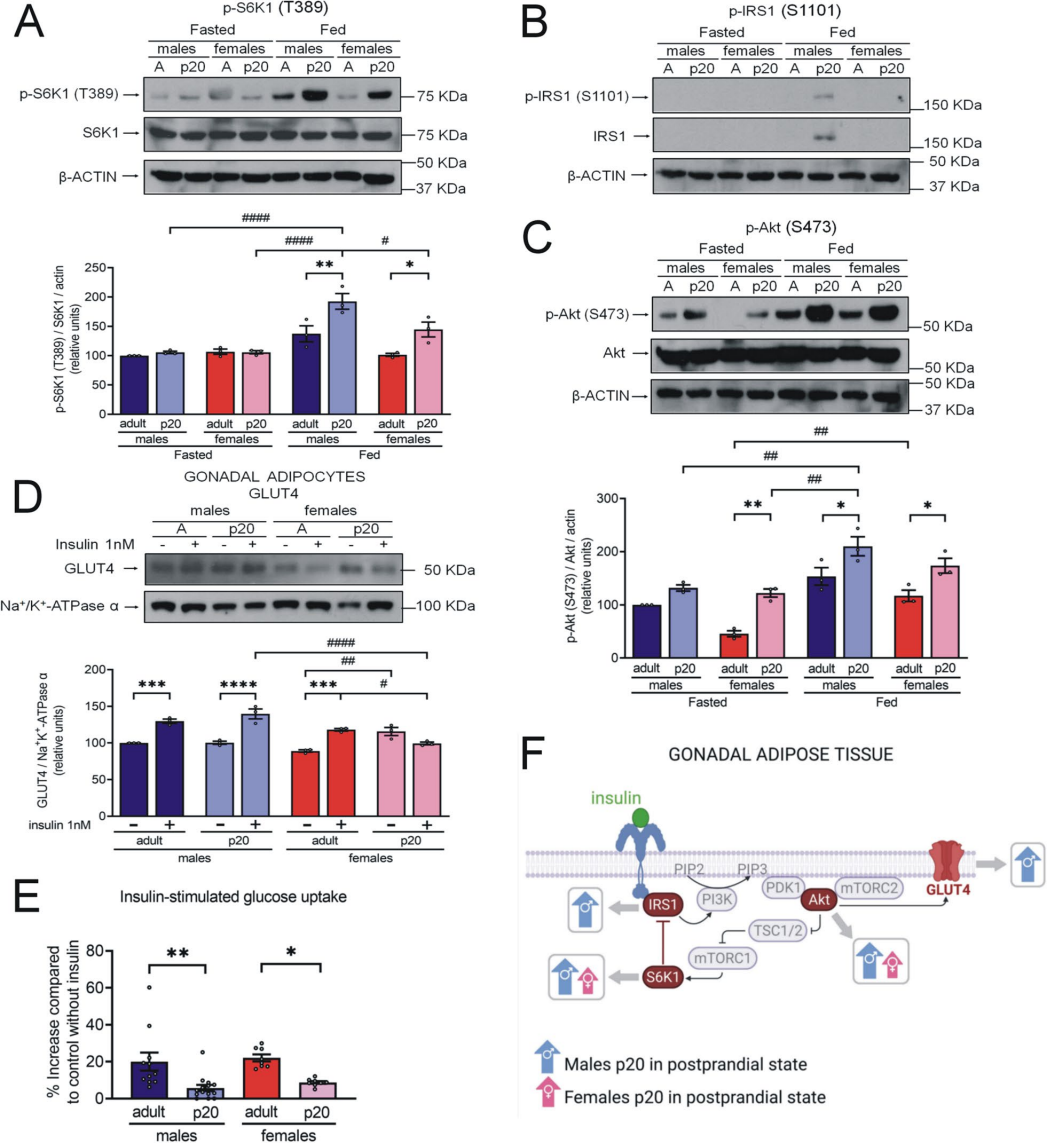
Sex- and age-related differences in the insulin signaling in gWAT.** a–c** Protein extract (100 μg protein) was separated by SDS-PAGE and subjected to Western blot analysis. We made tissue pools of 15 rats for p20 tissues and 5 rats for adult tissues. Results are product of three independent experiments. The bars represent the mean ± S.E.M. *P < 0.05, **P ≤ 0.005 compared to their control group by two-way ANOVA with post hoc Tukey's test; ^#^*P* < 0.05, ^##^*P* ≤ 0.007, ^####^*P* < 0.0001 compared between sex and fasting vs fed state by two-way ANOVA with post hoc Tukey's test; **d** Adipocytes from gonadal depot were isolated with tissue digestion by collagenase P and incubated with insulin 1 nM or DMSO 0.1% for 30 min. Plasma membrane proteins were labeled with sulfo-NHS-SS-biotin. Labeled proteins (60 μg protein) were separated by SDS-PAGE and subjected to analysis by Western blot. Results are product of three independent experiments. The bars represent the mean ± S.E.M. ***P ≤ 0.0005, ****P < 0.0001 compared to their control group by two-way ANOVA with post hoc Tukey's test; ^#^*P* < 0.05, ^##^*P* < 0.005, ^####^*P* < 0.0001 compared between sex and without stimulus vs with stimulus by two-way ANOVA with post hoc Tukey’s test; **e** Insulin-stimulated glucose uptake assay, gWAT (50 mg) was incubated for 30 min with 2-NBDG glucose 100 mg/mL (control condition) and with 2-NBDG glucose 100 mg/mL and insulin 100 nM (stimulating condition), n = 10 rats in each group. The bars represent the mean ± S.E.M. *P < 0.05, **P < 0.005 compared to their control group and compared between sex by two-way ANOVA with post hoc Tukey's test; **f** Summary of sex-related differences in the IR mechanism of PI3K/Akt signaling pathway in gWAT of p20 rats during postprandial state. Created with GraphPad Prism V 9.3.1 (471) and BioRender.com

**Fig. 4:**
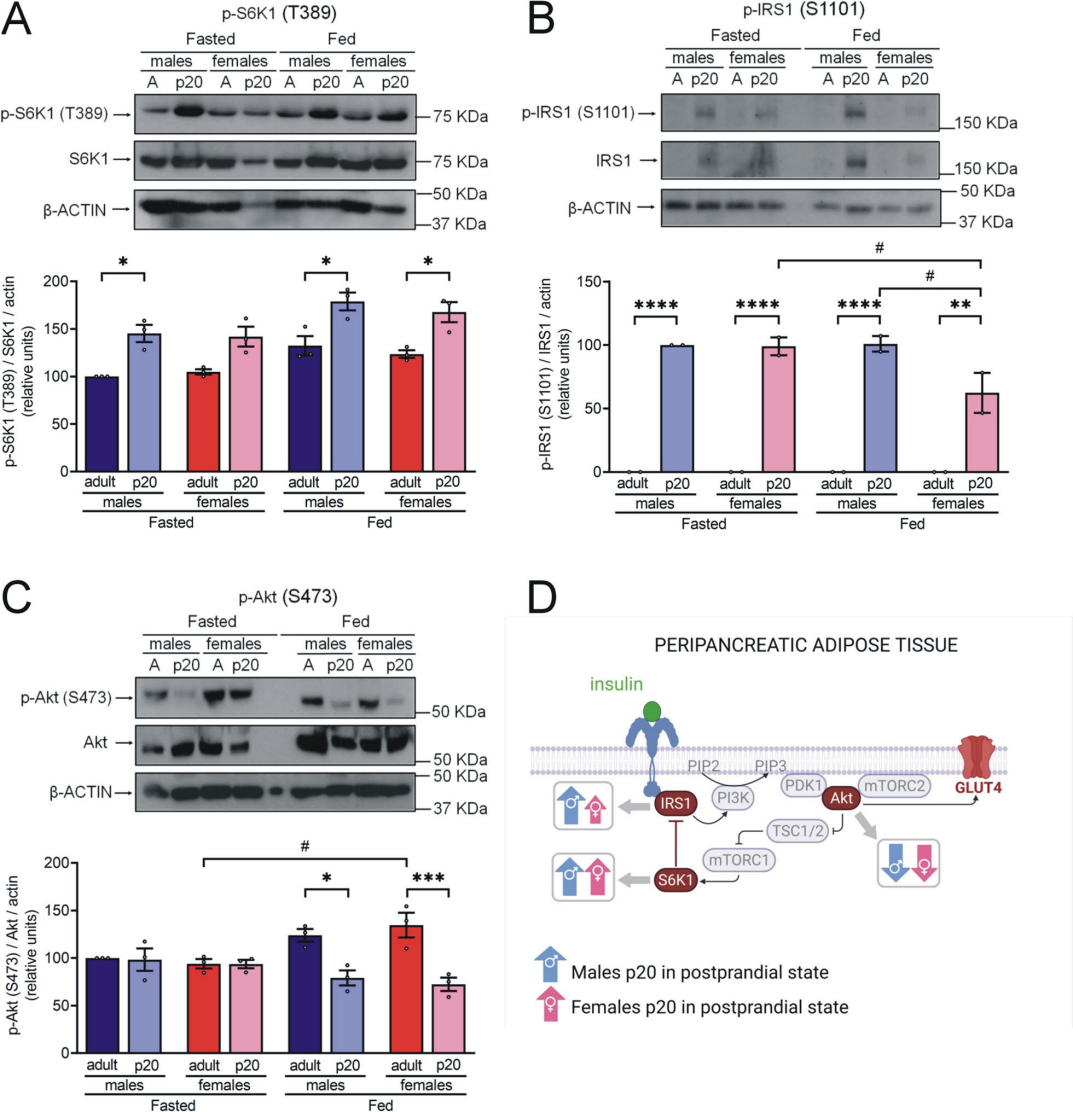
Sex- and age-related differences in the insulin signaling in pWAT. **a**–**c** Protein extract (100 μg protein) was separated by SDS-PAGE and subjected to Western blot analysis. We made tissue pools of 15 rats for p20 tissues and 5 rats for adult tissues. Results are product of three independent experiments to p-S6K1 and p-Akt and two to p-IRS1. The bars represent the mean ± S.E.M. *P < 0.05, **P < 0.005, ***P = 0.0009, ****P < 0.0001 compared to their control group by two-way ANOVA with post hoc Tukey's test; ^#^*P* < 0.05 compared between sex and fasting vs fed state by two-way ANOVA with post hoc Tukey's test; **d** Summary of sex-related differences in the IR mechanism of PI3K/Akt signaling pathway in pWAT of p20 rats during postprandial state. Created with GraphPad Prism V 9.3.1 (471) and BioRender.com

- *S6K1 overactivation and IRS1 inhibition do not inhibit GLUT4 translocation in gWAT of p20 rats*
  We observed higher levels of p-S6K1(T389) (active form) in the gWAT of p20 rats compared to adult rats during the fed state (Fig. 3a). Higher levels of p-S6K1(T389) were observed in males compared to females. Levels of the IRS1 inhibited form were only detected in fed p20 male rats (Fig. 3b). We found higher levels of p-Akt(S473) in gWAT of p20 rats compared to adult rats (Fig. 3c).
  Moreover, we observed an increase in GLUT4 abundance in the membrane of adipocytes following insulin stimulation (1 nM) in all groups except p20 females (Fig. 3d). Notably, GLUT4 levels following stimulation with insulin were higher in p20 males than in p20 females.
  Insulin-stimulated glucose uptake assay was performed on gWAT. Despite the increase of membrane GLUT4 in p20 adipocytes, levels of glucose uptake were lower than in adult adipocytes (Fig. 3e). Figure 3f shows a summary of these results.
- *Decreased Akt activation may be related to S6K1 overactivation and IRS1 inhibition in pWAT of p20 rats*
  We observed higher levels of p-S6K1(T389) and p-IRS1(S1101) in the pWAT of p20 rats compared to pWAT of adult rats of both sexes under both fasting and fed conditions (Fig. 4a, b). Interestingly, IRS1 and p-IRS1(S1101) were undetectable in adults. In contrast to gWAT, lower postprandial levels of p-Akt(S473) were observed in pWAT of p20 rats than in pWAT of adult rats (Fig. 4c).
  No differences were detected in pWAT levels of p-S6K1(T389) or p-Akt(S473) between males and females. However, postprandial levels of p-IRS(S1101) were lower in p20 females than in p20 males. Figure 4d shows a summary of these results.
- *S6K1 is overactivated in the skeletal muscle of p20 rats while Akt phosphorylated remains decrease.*

**Fig. 5:**
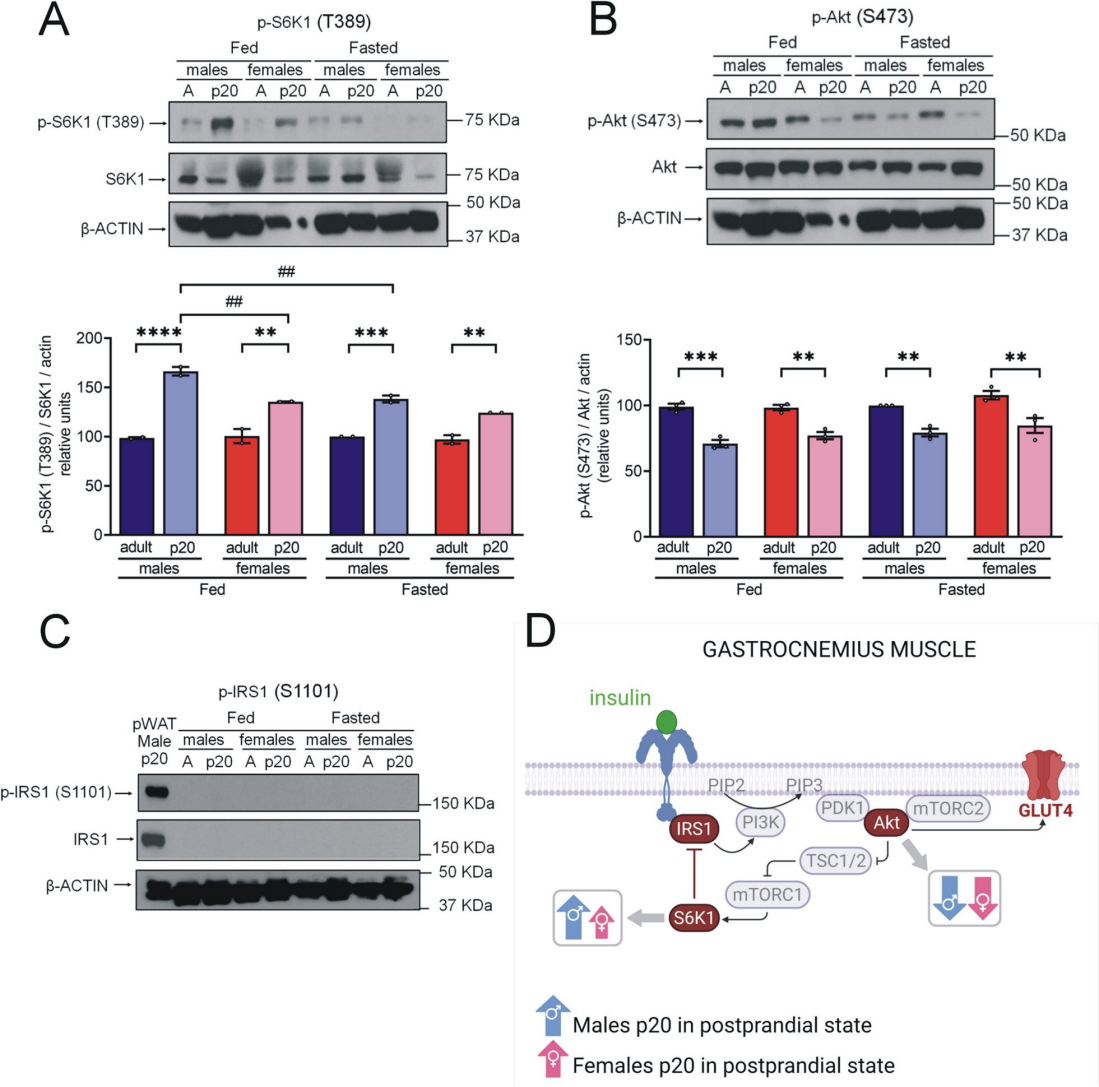
Sex- and age-related differences in the insulin signaling in gastrocnemius muscle. **a**–**c** Protein extract (60 μg protein) was separated by SDS-PAGE and subjected to Western blot analysis. We made tissue pools of 15 rats for p20 tissues and 5 rats for adult tissues. Results are product of two independent experiments to p-S6K1 and three to p-Akt. The bars represent the mean ± S.E.M. **P ≤ 0.009, ***P ≤ 0.001, ****P < 0.0001 compared to their control group by two-way ANOVA with post hoc Tukey's test; ^##^*P* ≤ 0.007 compared between sex and fasting vs fed state by two-way ANOVA with post hoc Tukey's test; **d** summary of sex-related differences in the IR mechanism of PI3K/Akt signaling pathway in gastrocnemius muscle of p20 rats during postprandial state. PAT: peripancreatic adipose tissue. Created with GraphPad Prism V 9.3.1 (471) and BioRender.com

We analyzed p-S6K1(T389) in the gastrocnemius muscle of both p20 and adult rats. We observed higher levels of p-S6K1(T389) in the muscle of fasted and fed p20 rats compared to adults, particularly in males (Fig. 5a). Interestingly, we observed that Akt activation is lower in p20 rats’ muscle than in adults under fasting and fed conditions (Fig. 5b). However, no levels of IRS1 or p-IRS1(S1101) in the gastrocnemius muscle could be detected (Fig. 5c). Figure 5d shows a summary of these results.

In general, the results of WAT and muscle suggest that changes in insulin signaling in p20 rats are more robust in males than in females.

## Discussion

Glucose homeostasis involves a complex relationship between pancreatic beta cells and peripheral tissues and their regulation by the central nervous system. Impaired metabolic responses of organs to insulin generate IR [1]. During some stages of development, this condition arises and is typically transient [19, 22]. However, the molecular mechanisms involved in the physiological IR are largely unknown. We studied a critical developmental window in *Wistar* rats, around weaning, at postnatal day 20 (p20) to characterize the IR that occurs in healthy and growing animals.

The impairment of the response of target organs to insulin involves various cellular mechanisms. Elevated fasting glucose and insulin levels are characteristic of obesity-induced IR. Hyperinsulinemia favors a sustained stimulus to insulin signaling pathway that drives metabolic actions, progressively inducing insulin desensitization [5]. The availability of insulin receptors in the membrane varies due to their internalization, degradation or recycling [31, 32]. In addition, the insulin receptor can suffer proteolytic cleavage, releasing its ectodomain and interrupting insulin signaling [33]. On the other hand, in the proximal signaling phase, from the receptor to Akt, some kinases and phosphatases act on critical nodes inhibiting the insulin signaling in a nonregulated manner [1, 5, 32].

In p20 rats, we observed high fasting and postprandial blood glucose and insulin levels. The ITT showed that target organs have lower glucose internalization in response to insulin in p20 rats. In the IPGTT, we observed that p20 rats are less tolerant to glucose; this response is related to the functional maturation of pancreatic beta cells during the early development [22, 24]. These data suggest that an imbalance of glucose homeostasis may be due to systemic insulin resistance in p20 rats.

In adults, we identified differences in insulin sensitivity between males and females. We found higher fasting blood glucose levels in females. This observation contrasts with some studies that report higher fasting glucose in male adult rats than in females [34]. We observed, that female adult rats are less sensitive to insulin than males, contrary to what previously reported [35].

The differences between our results and others may be related to the fluctuations of female sex hormones along the estrous cycle of adult rats. The estrous cycle of rats lasts between 4 and 5 days. High estradiol and progesterone levels are observed during the proestrus phase, while in metestrus, diestrus, and estrus concentrations of these hormones are relatively low [36]. Estrogens regulate rodents’ energy balance, body weight, fat distribution, and appetite [37]. Estrogens increase insulin sensitivity in tissues and upregulate insulin transcription in pancreatic beta cells as well as GLUT4 synthesis in adipose tissue and muscle [17]. Female sex hormones play a fundamental role in the sexual dimorphism of insulin sensitivity during adulthood. Therefore, we consider that variations in the concentrations of these hormones may be related to changes in insulin sensitivity in adult female rats. Further research is needed due to the lack of murine models to assess the role of hormones in central and peripheral insulin sensitivity.

Obesity-induced inflammation causes IR [1]. Pro-inflammatory cytokines, such as TNF-α and IL-1β, activate kinases such as JNK, IKK, or PKCs that inhibit insulin signaling [28, 38]. We determined TNF-α, IL-1β, and GH to further analyze mechanisms that could be involved in the development of IR at p20. We identified lower plasma levels of TNF-α, and IL-1β in p20 than in adult rats, suggesting that the IR observed in p20 rats is not related to inflammation-induced mechanisms. In addition, female adult rats showed lower levels of TNFα and higher levels of IL-1β than adult male rats, as reported in previous studies [39].

On the other hand, insulin and GH are counteracting hormones; both regulate energy metabolism and cell growth through different signaling pathways that converge and regulate each other [11, 12]. Previous works in different animal models have observed that high levels of GH induce IR by inhibiting PI3K/Akt signaling pathway. It has been demonstrated that GH signaling decreases IRS activity via Janus kinase 2 (JAK2)/signal transducer and activator of transcription 5 (STAT5)/suppressor of cytokine signaling (SOCS) kinases [11]. Furthermore, in vivo and in vitro studies have demonstrated that prolonged exposure to insulin inhibits GH signaling [12]. However, whether both GH and insulin upregulate mTORC1/S6K1 signaling during accelerated growth periods is still unknown.

During the lactation period of the rat, serum GH levels decrease gradually, but after weaning, they rise to reach a plateau at day 80 [40]. We found that p20 rats show lower blood levels of GH than adult rats, in which we found very high levels. The last could be due to the circadian release of GH, which reaches elevated levels (around 60 ng/mL) during sleeping periods and could increase in response to prolonged fasting [41]. The daytime at which the experiments were performed and the 12-h fasting period could have probably induced an increase in GH levels of adult rats in our experiments. We consider that further research to assess the role of GH in the regulation of insulin signaling during the weaning period is needed.

Insulin signaling is regulated positively and negatively depending on the function and metabolic needs of each tissue. However, this regulation is lost when obesity and inflammation exist. Unregulated inhibition may involve a constitutive activation of S6K1 that could inhibit IRS1 signaling and promote its degradation resulting in IR and the development of metabolic diseases [42]. Several research concerning these mechanisms have identified multiple inhibitory phosphorylation sites in IRS1 that may be involved in the unregulated inhibition of insulin signaling [7, 43–46]. In addition, it has been suggested that the hyperphosphorylation of S6K1 observed in different IR models may result in the inhibition of both PI3K and Akt kinases [8, 9].

Although the mechanism of S6K1 overactivation and IRS1 inhibition have been described in pathological contexts, we explored these mechanisms in healthy rats, because p20 is a stage of accelerated growth in which S6K1 is overactivated due to the increase in protein synthesis [6, 20]. In addition, during the weaning period, the dietary transition from maternal milk to carbohydrate-rich food constitutes a driving force in the functional maturation of pancreatic beta cells and insulin target organs [22, 47, 48]. The metabolic adaptations in the mTORC1/S6K1 axis during accelerated growth and dietary transition [49] may play a role in the development of physiologic IR.

Our results suggest that overactivation of S6K1 and IRS1 phosphorylated at S1101 may not be involved in the inhibition of Akt on gWAT of male p20 rats. Moreover, GLUT4 translocation to the membrane was maintained, probably due to a conserved activity of the Akt/TBC1D4/GLUT4 pathway in adipocytes. However, insulin-stimulated glucose uptake is lower in p20 rats, this may be related to underlying mechanisms that regulate GLUT4 activity —for example, other related signaling pathways such as the Rho GTPase TC10α pathway—. It has been demonstrated that TC10α is activated upon insulin stimulation in an Akt-independent manner. Moreover, the knockdown of TC10α by siRNA results in a reduced insulin-stimulated glucose uptake in adipocytes [1].

On the other hand, previous studies have reported that colocalization of GLUT4 and clathrin-coated pits at adipocyte membrane, is promoted by the adaptor protein AP2, which regulates GLUT4 function and its distribution in the cell membrane [1, 50]. Recently, Gao and collaborators [50] reported that IR induces clustering of GLUT4 at the membrane, decreasing the efficiency of glucose transport into the cells. Functional maturation of the insulin-target organs occurs during a critical developmental window around p20. According to our results, insulin stimulation promotes GLUT4 translocation to the membrane. However, the transporter shows a low activity in adipocytes of gWAT from p20 rats. Our results suggest that the maturation of protein machinery involved in regulating the distribution and function of GLUT4 at adipocyte plasma membrane occurs during this critical developmental window. We propose more research to explore these mechanisms during this stage of development.

In addition, we identified that the insulin signaling pathway shows sexual dimorphism at gWAT of p20 rats. The levels of p-S6K1(T389), p-IRS1(S1101), p-Akt(S473) and GLUT4 abundance at the membrane are higher in male than in female rats.

Furthermore, we identified S6K1-mediated IRS1 inhibition and low levels of p-Akt(S473) in pWAT of p20 rats of both sexes. The reduction in Akt activation may contribute to the systemic IR that arises during this stage. Unfortunately, this fat depot is very scarce in p20 rats, so we could not determine GLUT4 activity in it. In contrast to gWAT, we observed high levels of p-IRS1(S1101) in pWAT of p20 rats of both sexes in fasting and fed states. Several studies have suggested that IRS1 is the principal isoform in WAT in adult animals. However, we did not detect levels of this isoform in the pWAT of adult rats. IRS1 protein levels are probably below the detection threshold at the conditions used in the assays. Further research is needed to determine whether this IRS isoform is the most abundant at this age under these conditions.

These results support previous evidence of metabolic differences between fat depots [51]. gWAT may show a more robust paracrine response to sex hormones or sex-related genetic components. During the early stage of development, the crucial role of sex chromosomes in adiposity is evident prior to the exposure to gonadal hormones [52]. In pWAT, insulin action may be different, probably due to its proximity to the pancreas and local insulin levels [29].

Although insulin sensitivity of adipose tissue is a determining factor in systemic IR development [53], muscle insulin sensitivity also plays an important role. The skeletal muscle shows high levels of insulin-dependent glucose uptake, and the sex-dimorphism observed in postprandial glucose levels exhibit differences in glucose internalization and metabolism in this tissue [30]. We found overactivation of S6K1 and lower p-Akt(S473) levels in muscle of p20 rats in both fasting and fed conditions, and we detected neither IRS1 nor p-IRS1(S1101) in this tissue. Furthermore, according to our results, maybe a low activation of Akt may not be related to the overactivation of S6K1 and the resulting inhibition of IRS1. However, Akt is a critical node of regulation in metabolic actions of insulin, and its inhibition may contribute to IR observed in p20 rats. We consider important exploring the role of IRS2 isoform in muscle, which could play a relevant role in the metabolism of this tissue at the age and feeding conditions studied in this work.

Although, we observed high levels of the activated form of S6K1 in adipose tissue and skeletal muscle of p20 rats, particularly in males, its inhibitory effect on IRS1 by phosphorylation at S1101 is not associated with a reduced activity of Akt in all tissues; this only occurs in pWAT. Activation and inhibition of Akt in gWAT and skeletal muscle may be related to the activity of other regulatory kinases or phosphatases and not necessarily results from the S6K1-induced IRS1 inhibition. Several studies have observed that insulin-induced Akt phosphorylation is normal in some types of IR, despite IRS1 downregulation, which suggests that defects in IRS1 activation may induce IR in an Akt-independent fashion [3].

Our results indicate that the mechanism that involves S6K1-induced inhibition of p-IRS1(S1101) is tissue-specific and partially contributes to systemic physiological IR in this critical developmental window of the rat. Several works using animal models of obesity, have observed high levels of p-S6K1(T389) in the liver, skeletal muscle, and adipose tissue during fasting states. They are further increased after insulin stimulation. Constitutive activation of S6K1 promotes hyperphosphorylation and degradation of IRS1 [7, 8]. The configuration settings of phosphorylated sites [46] and other post-translational modifications determine protein function [44]. IRS1 phosphorylation at S1101 by S6K1 could play an important role in metabolic stress mechanisms and contribute to the development of IR; this phosphorylation has been cataloged as an early event in the desensitization of insulin signaling in pathological contexts [7, 46].

There is still considerable uncertainty about sexual dimorphism in the early stages of development, where the role of sex hormones is not present, and differences in insulin sensitivity and metabolism reported in postnatal development may be related to changes in gene expression observed along the transition from intrauterine to postnatal life [16]. During early postnatal life, changes in each organ are critical in their development and are highly susceptible to exogenous stimuli. An imbalance in their regulatory mechanisms may promote metabolic alterations that persist until adulthood and increase the risk of developing metabolic diseases. We identified differences in insulin signaling between males and females in several tissues at p20. However, these differences are not evidenced in systemic IR observed in these rats, suggesting that metabolism in each organ and its relationship with glucose homeostasis is essential during this critical developmental window.

Assessing these mechanisms and their sex-dimorphism is essential to understanding insulin actions and their pathophysiological role in developing metabolic dysfunction. This work sets a precedent in identifying therapeutic targets of metabolic diseases such as T2DM, in males as historically studied and in females.

## Conclusions

**Fig. 6:**
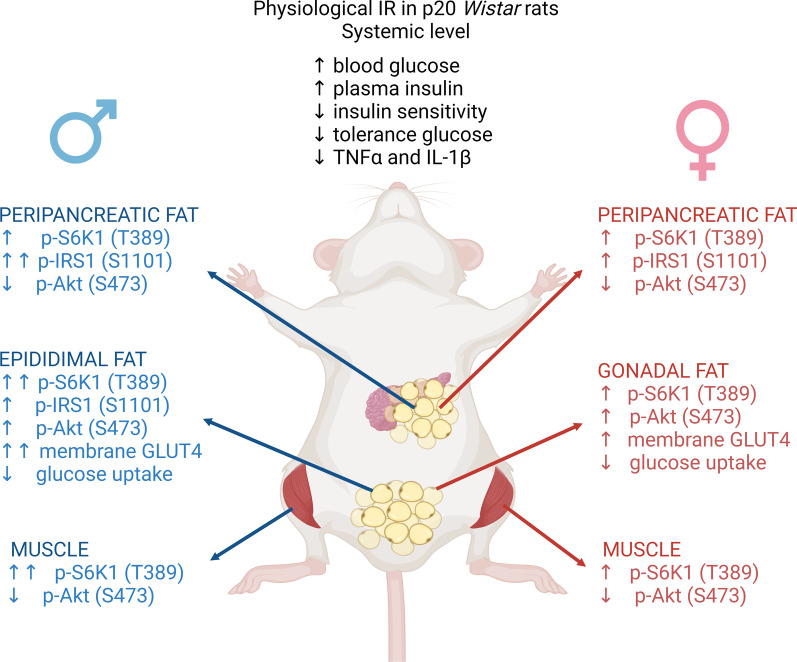
Physiological insulin resistance in p20 *Wistar* rats. Systemic insulin resistance is similar between males and females p20 rats. However, there are differences in insulin signaling pathway proteins between the sexes, tissues and even in different depots of white adipose tissue

We characterized physiological IR observed at postnatal day 20, around weaning, in male and female *Wistar* rats. We found that both male and female p20 rats show insulin resistance with similar features. However, we identified sex-related differences in the activation of insulin signaling pathway proteins in different tissues and even in different depots of WAT (Fig. 6).

This study attempts to advance the comprehension of molecular mechanisms involved in physiological IR at p20 by comparing them with mechanisms described in pathological IR and considering the metabolic response of mTORC1/S6K1 to changes during this critical developmental window. Our data indicate that S6K1 overactivation and IRS1 phosphorylation at S1101 may modulate insulin signaling in a tissue-specific manner and contribute to systemic IR at this developmental stage.

The sexual dimorphism identified in molecular mechanisms of physiological IR during this stage contributes understanding the metabolic homeostasis in both sexes and highlights the importance of considering sex as a significant variable in experimental designs and treating of metabolic diseases.

## Supplementary Information

**Additional file 1**. Supplementary images from western blots.

## Data Availability

The dataset supporting the conclusions of this article is included within the article and its additional file.

## Abbreviations

Akt: Protein kinase B
AP2: Adaptor protein 2
AUC: Area under the curve
GH: Growth hormone
GLUT4: Glucotransporter 4
gWAT: Gonadal white adipose tissue
IKK: Inhibitor of nuclear factor-κB kinase
IL-1β: Interleukin-1β
IPGTT: Intraperitoneal glucose tolerance test
IR: Insulin resistance
IRS1: Insulin receptor substrate 1
IRS2: Insulin receptor substrate 2
ITT: Insulin tolerance test
JAK2: Janus kinase 2
JNK: Jun N-terminal kinase
mTORC1: Mammalian target of rapamycin complex 1
mTORC2: Mammalian target of rapamycin complex 2
p20: Postnatal day 20
PDK1: Phosphoinositide-dependent kinase-1
PI3K: Phosphatidylinositol 3-kinase
PIP3: Phosphatidylinositol 3,4,5-trisphosphate
PKCs: Proteins kinase C
pWAT: Peripancreatic white adipose tissue
S6K1: Ribosomal S6 kinase 1
siRNA: Small interfering RNA
SOCS: Suppressor of cytokine signaling
STAT5: Signal transducer and activator of transcription 5
T2DM: Type 2 diabetes mellitus
TBC1D4: TBC1 domain family member 4
TC10α: Human Rho family small GTP binding protein
TNFα: Tumor necrosis factor α
WAT: White adipose tissue
