# Supplementary material for: Sexual dimorphism in the molecular mechanisms of insulin resistance during a critical developmental window in Wistar rats
Source: Cell Commun Signal. 2022 Oct 12;20:154. doi: 10.1186/s12964-022-00965-6 (PMC9554987; doi:10.1186/s12964-022-00965-6)
Supplement: Supplementary file 2 — Additional file 1. Supplementary images from western blots. [file 12964_2022_965_MOESM2_ESM.pdf]

Tejido adiposo gonadal
21NOV2018

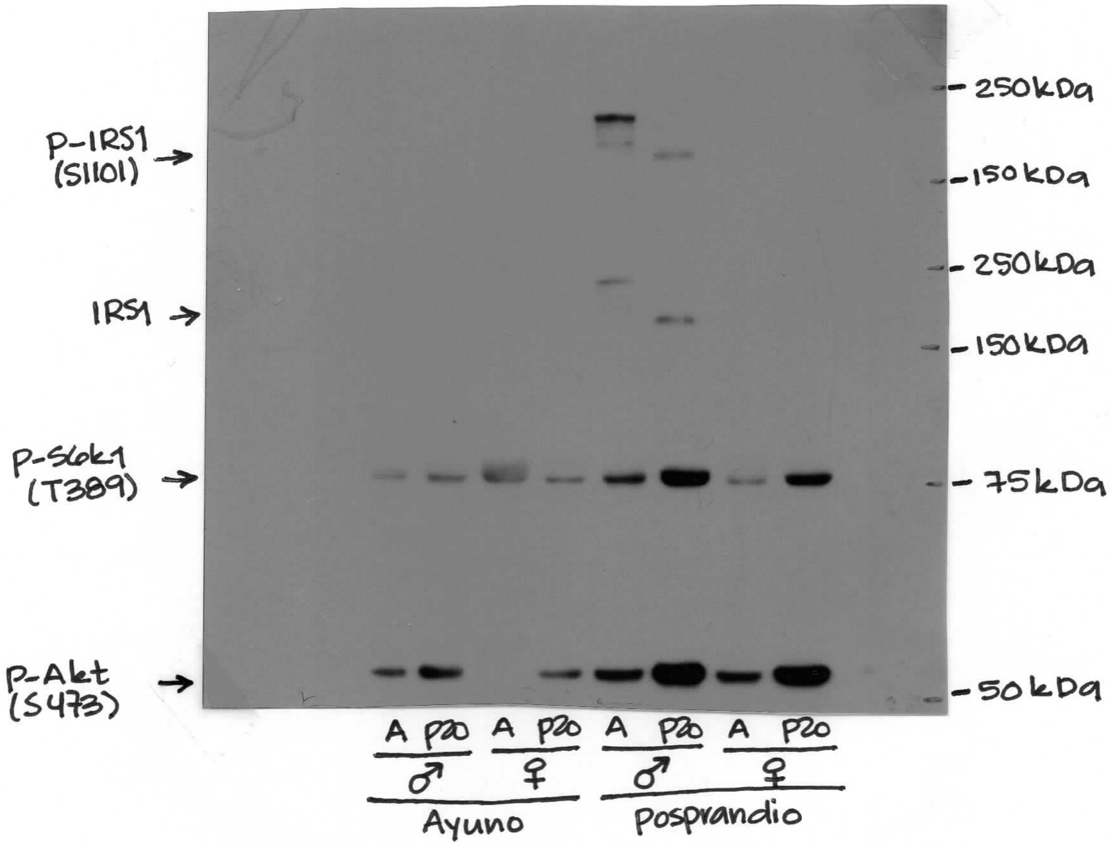

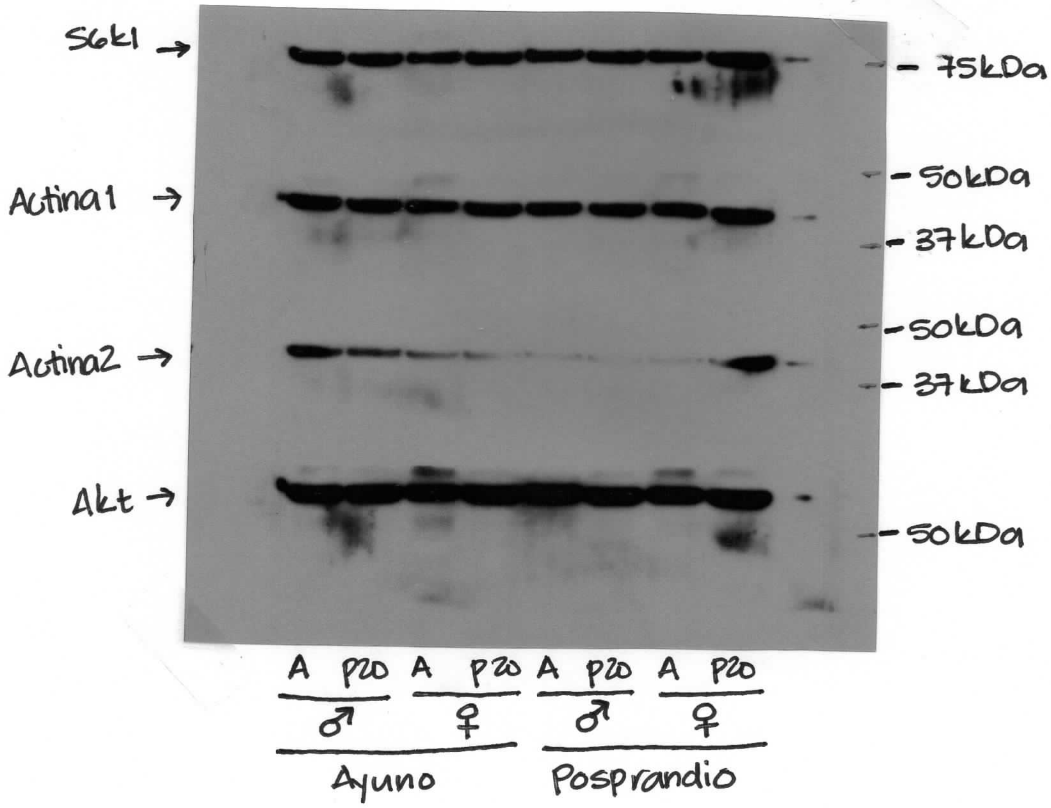

Tejido adiposo gonadal
21NOV2018

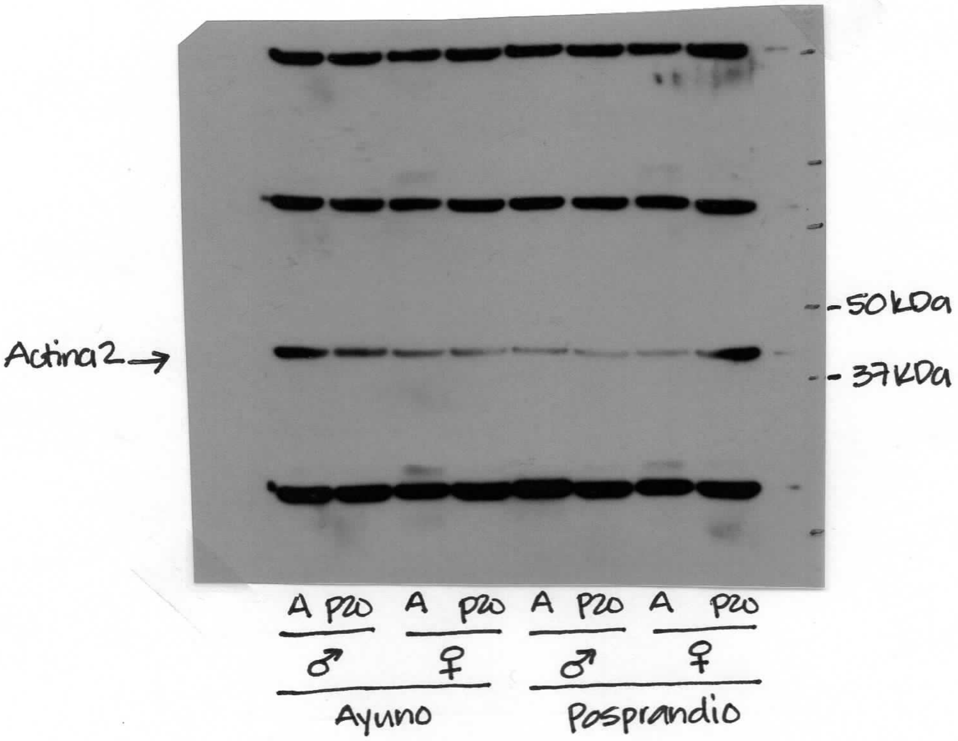

Tejido adiposo gonadal
17mayo 2018

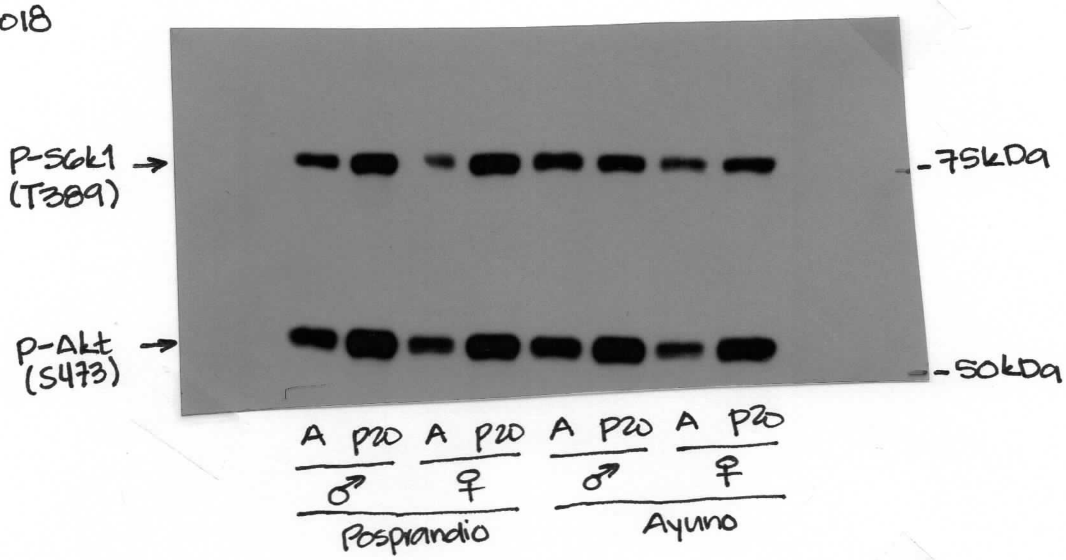

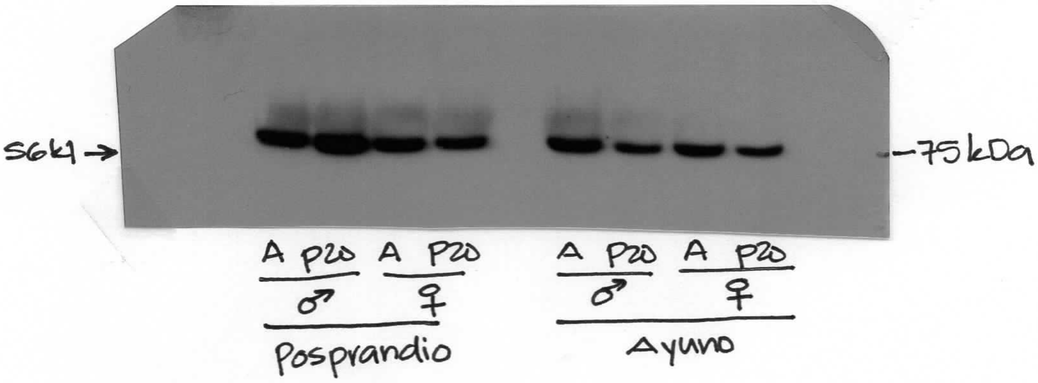

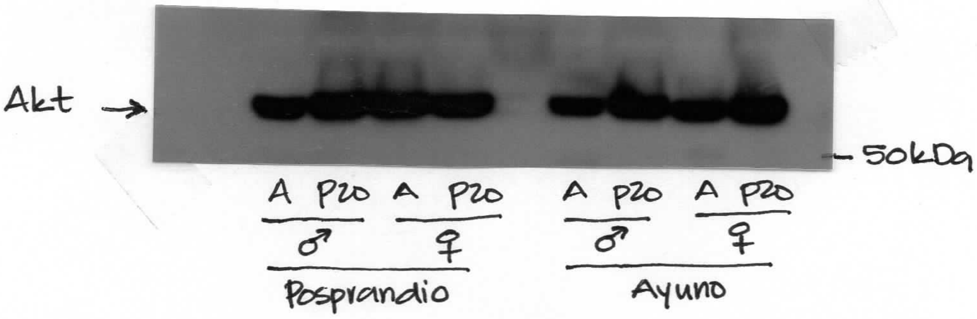

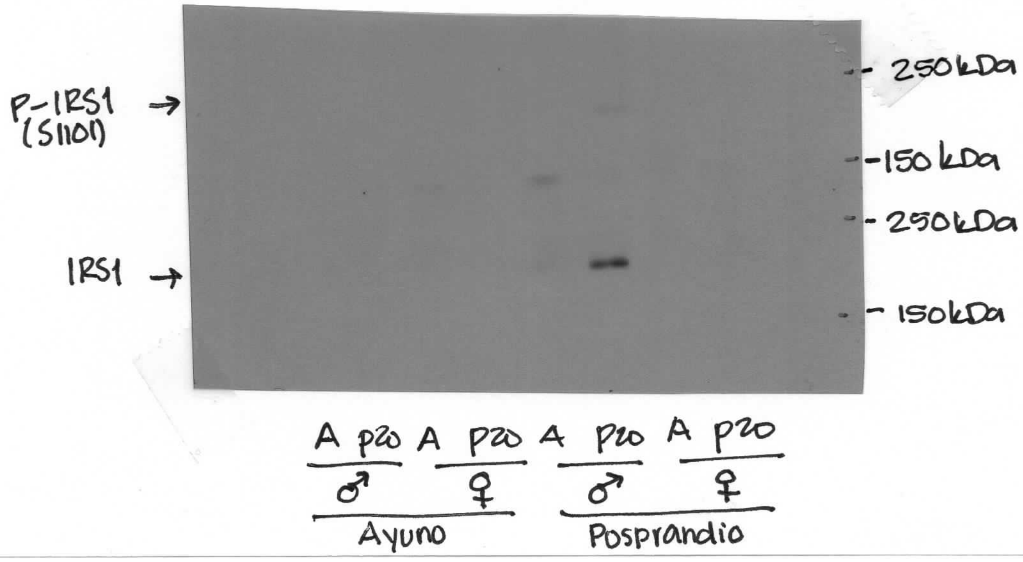

Tejido adiposo gonadal
17 Mayo 2018

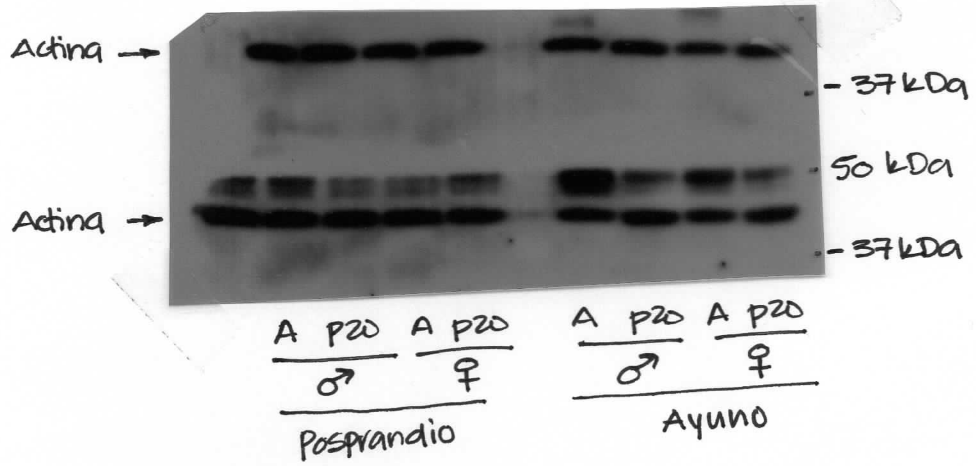

Tejido Adiposo Gonadal
25Oct2018

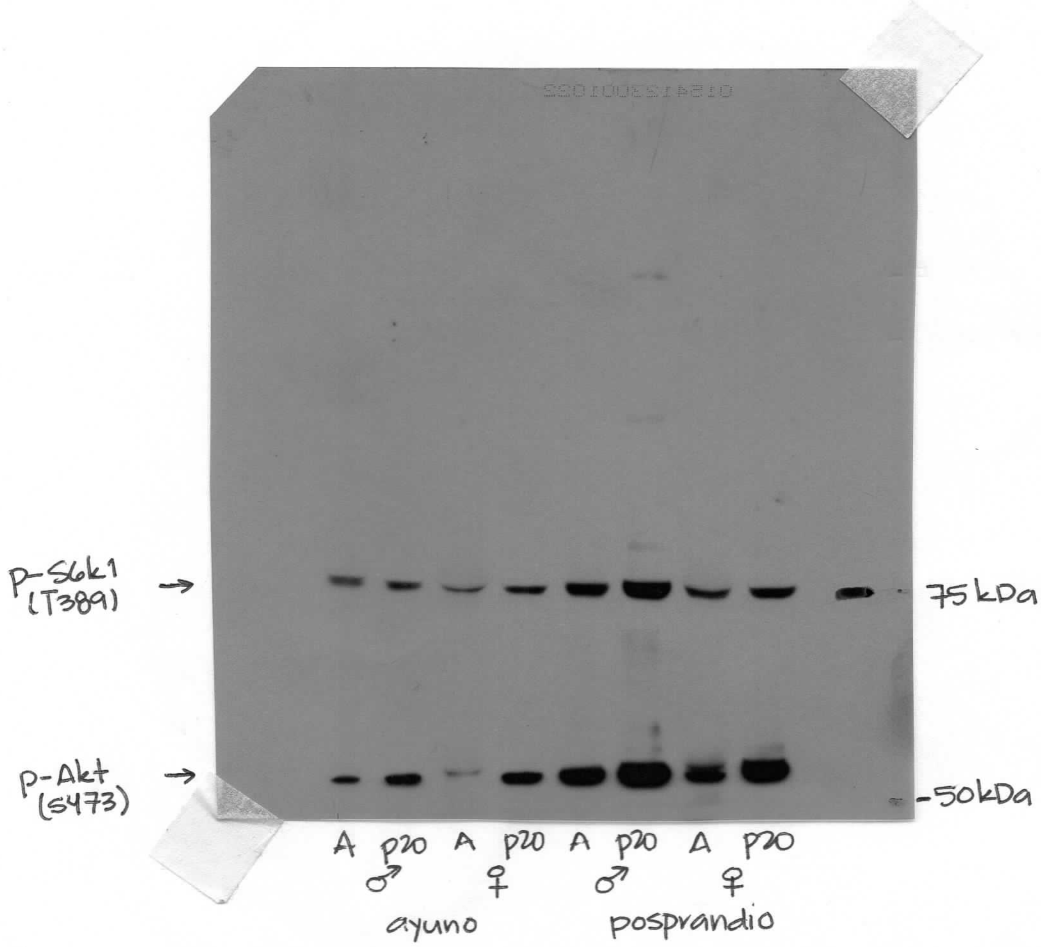

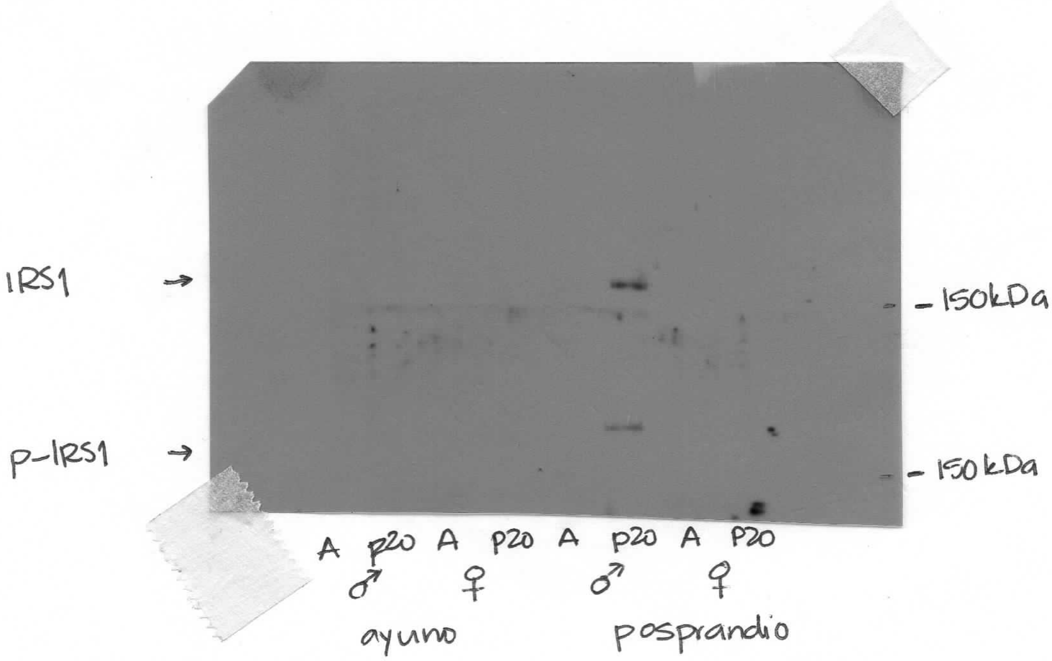

Tejido adiposo gonadal
25Oct2018

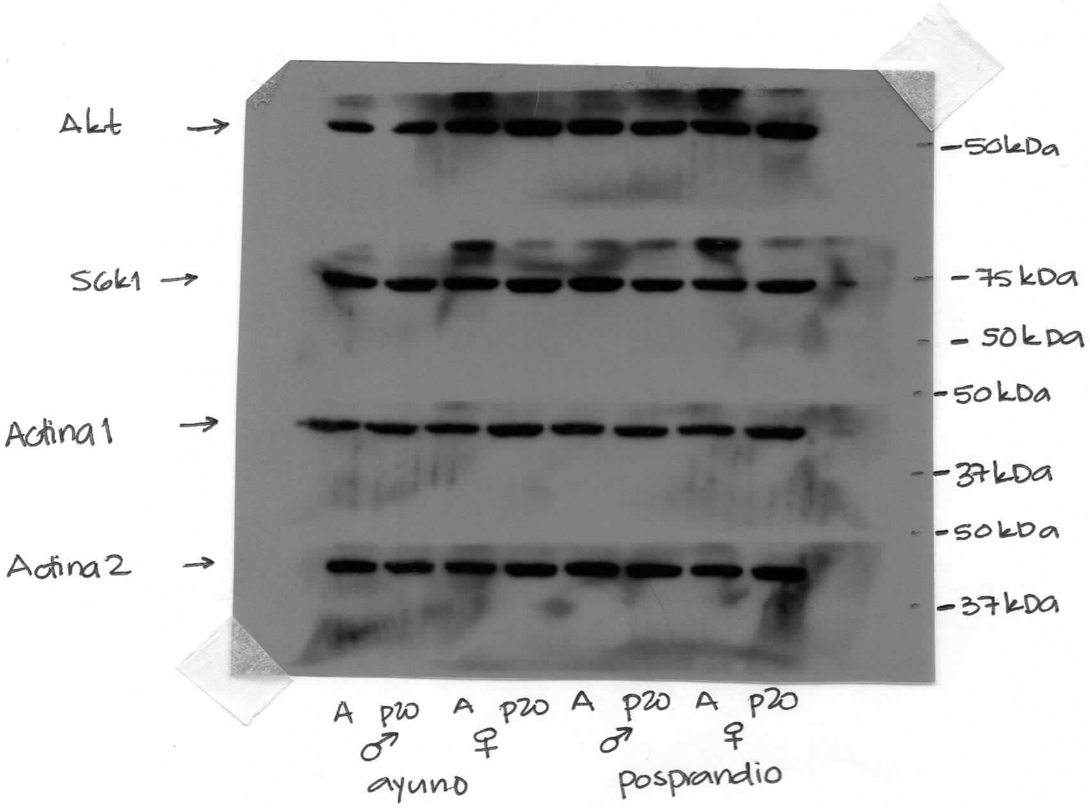

Tejido Adiposo Gonadal

extracto proteíco de células aisladas

17 Ene 2019

25 Ene 2019

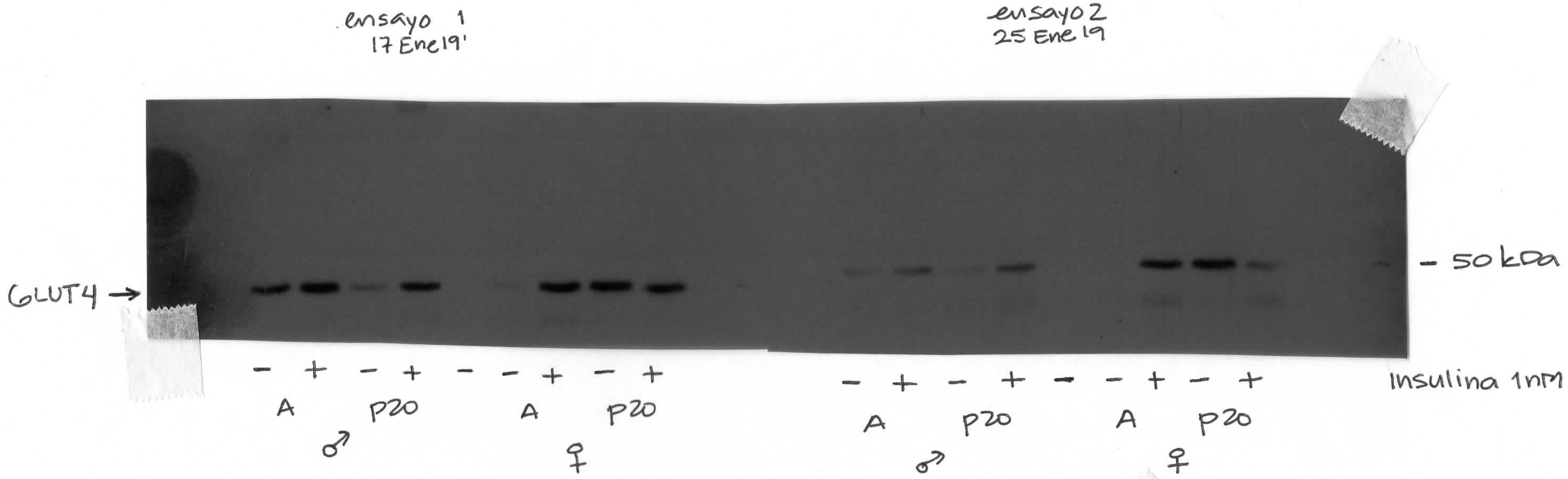

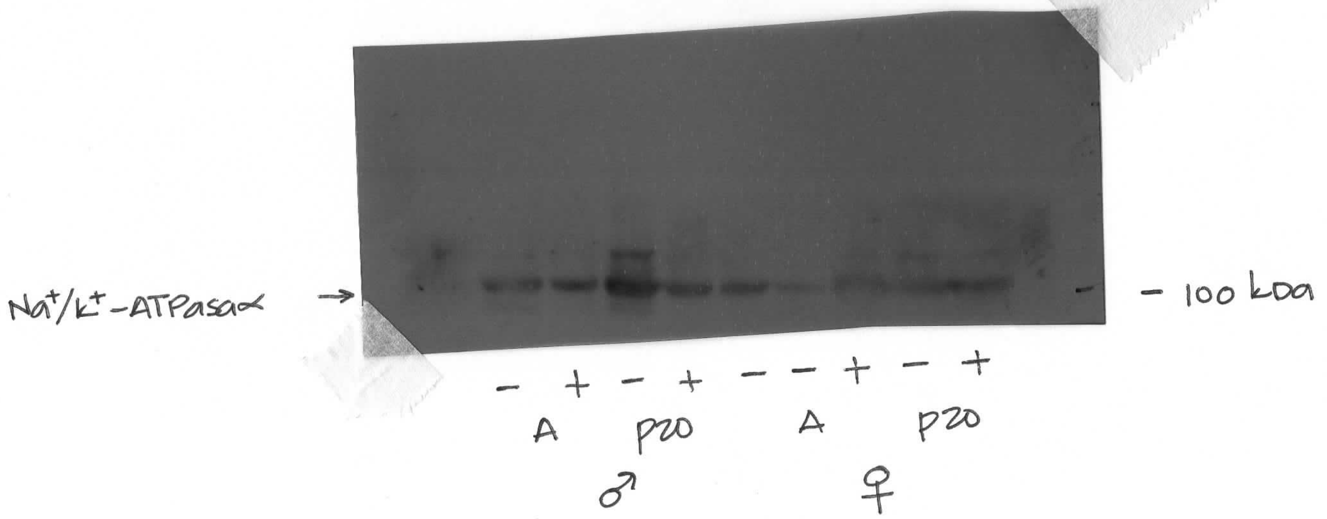

Tejido Adiposo Gonadal
extracto proteico de células aisladas
12 Marzo 2019

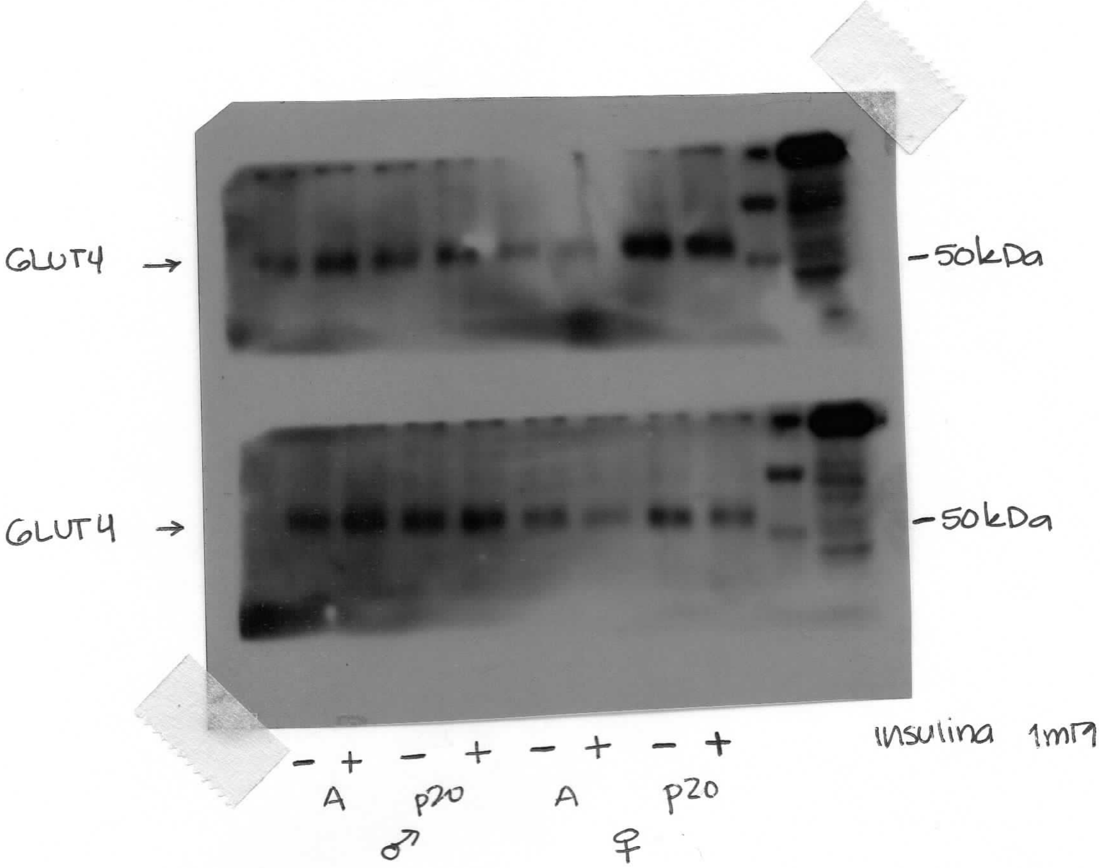

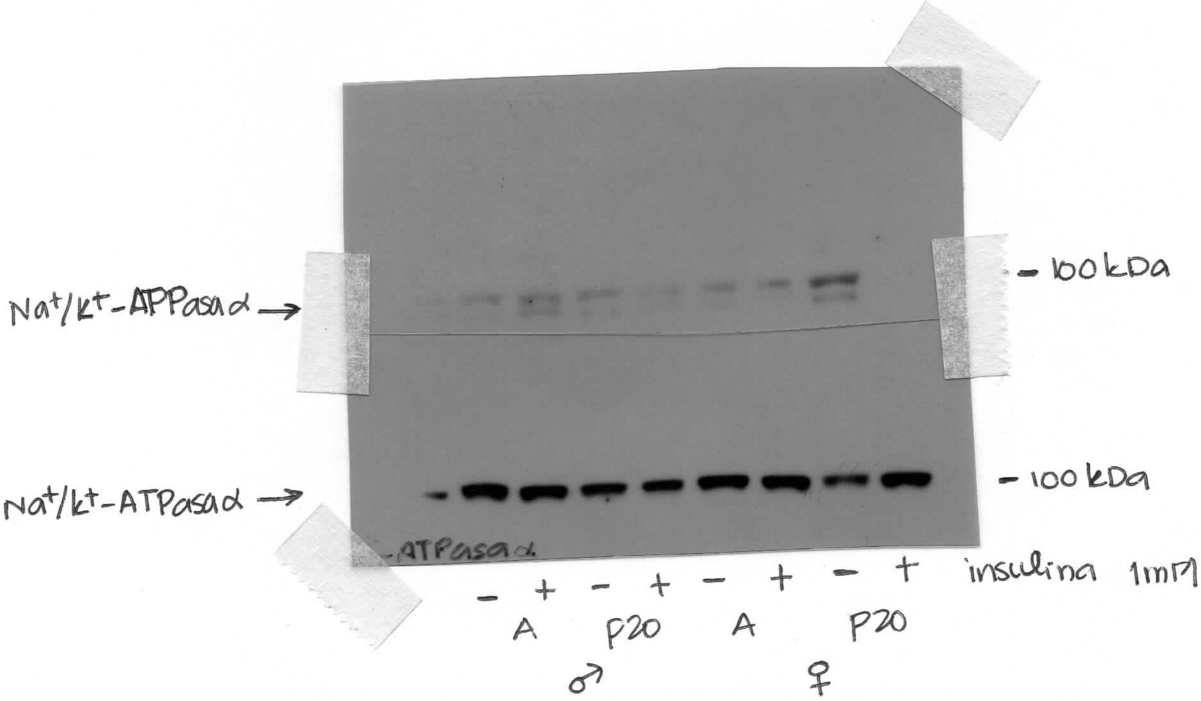

Tejido Adiposo Peripancreático
02Septiembre2019

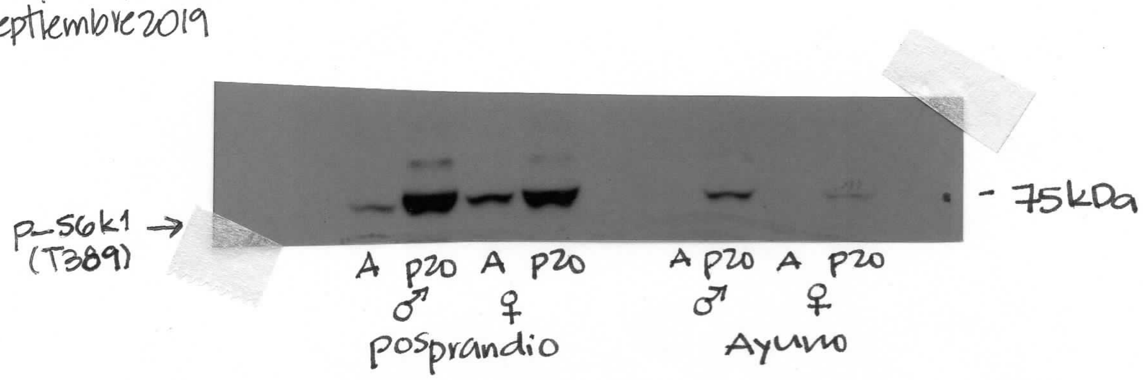

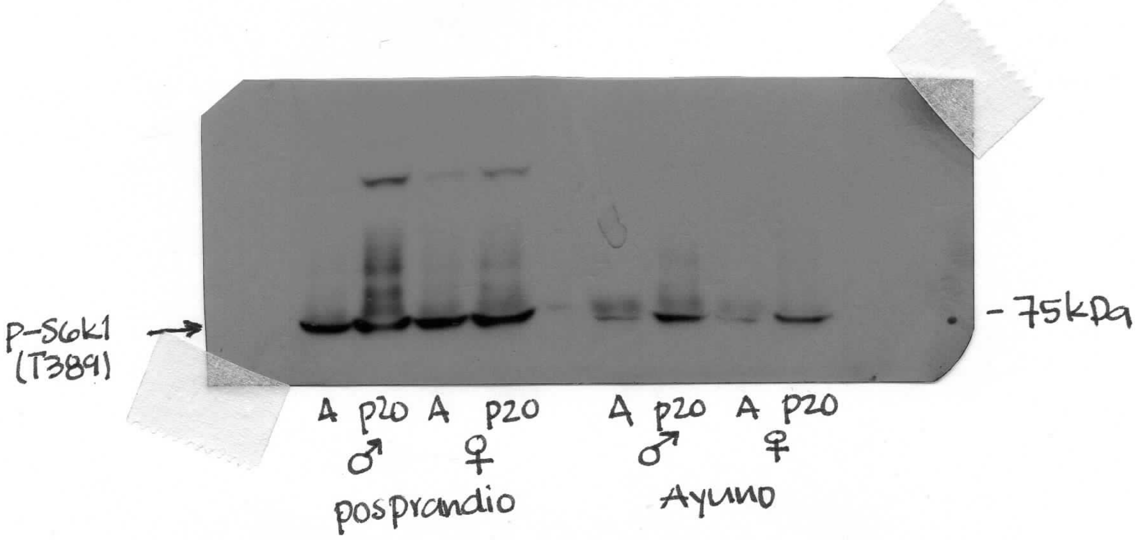

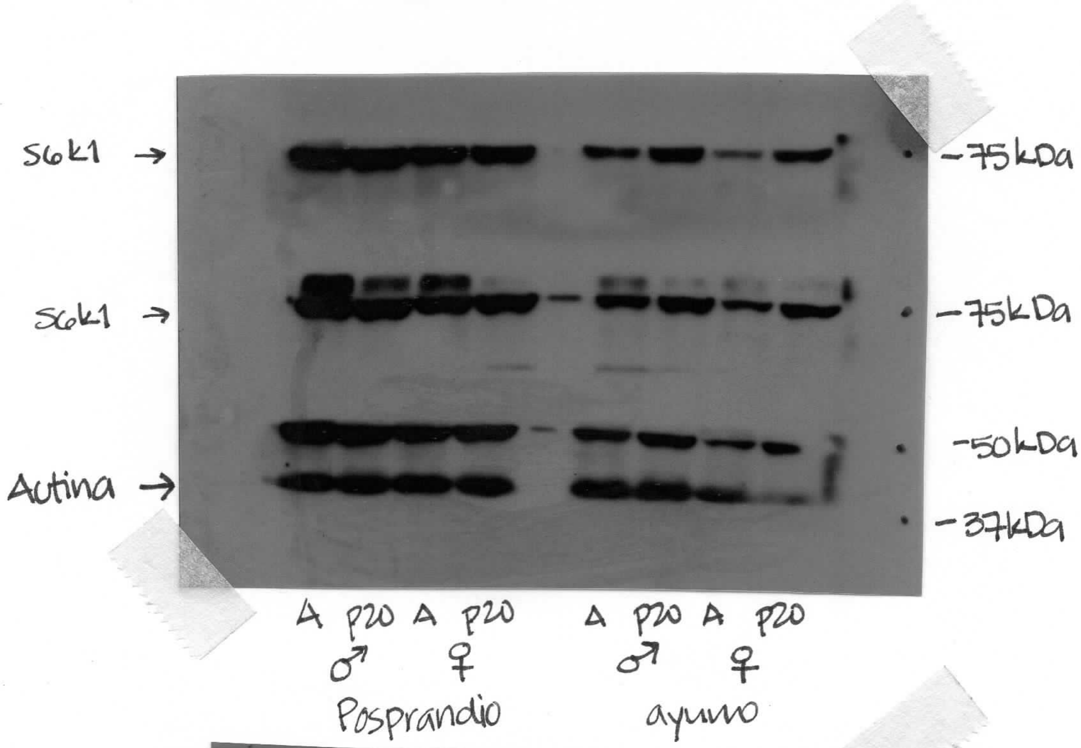

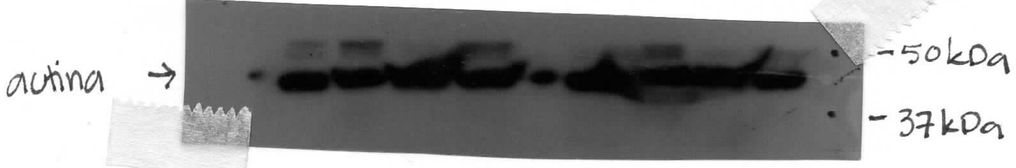

Tejido Adiposo Peripancreático
09NOV2017

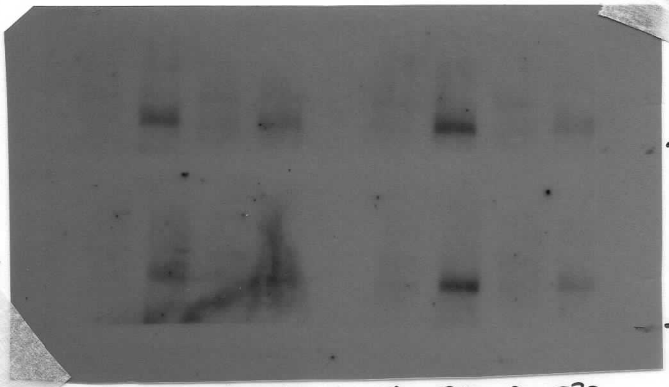

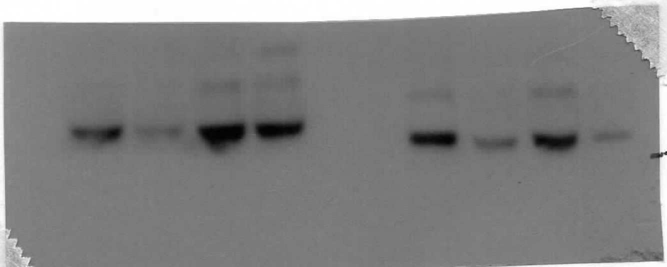

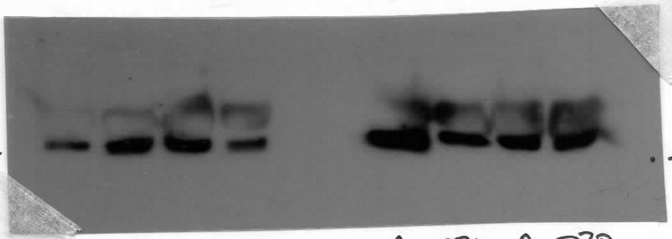

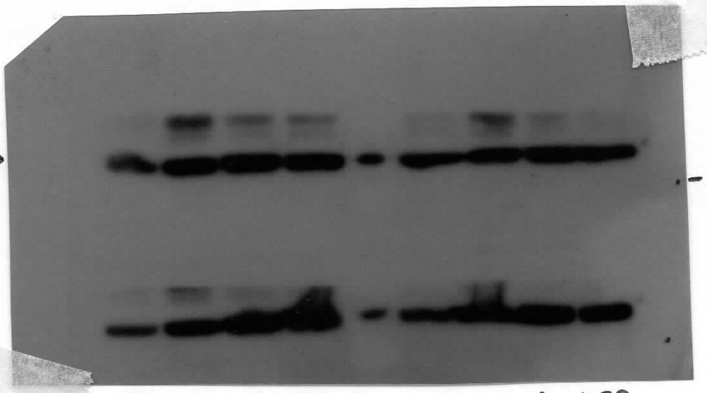

Tejido adiposo peripancreático
16 Mayo 2018
25 Mayo 2018

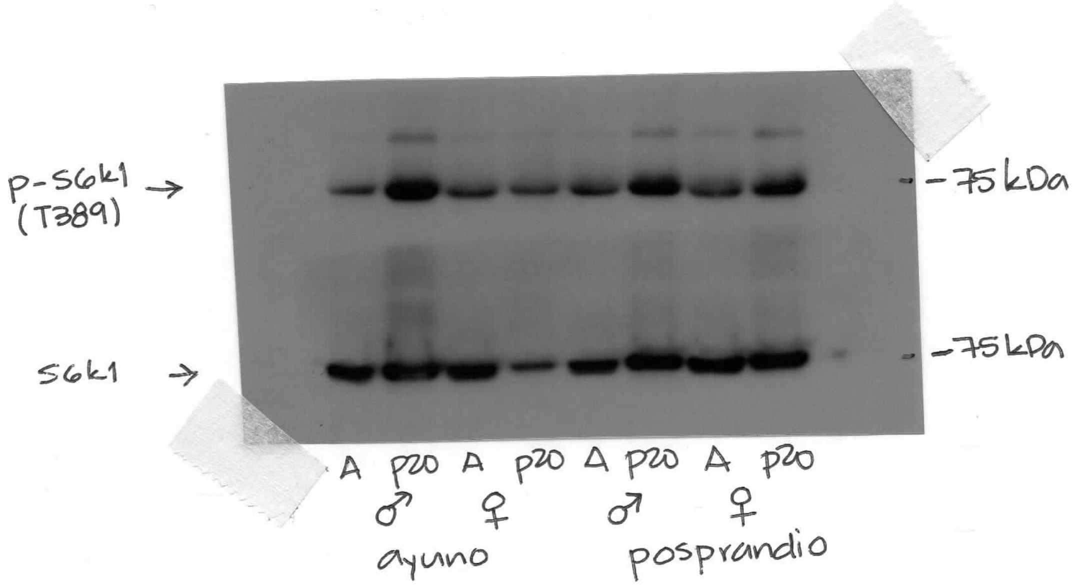

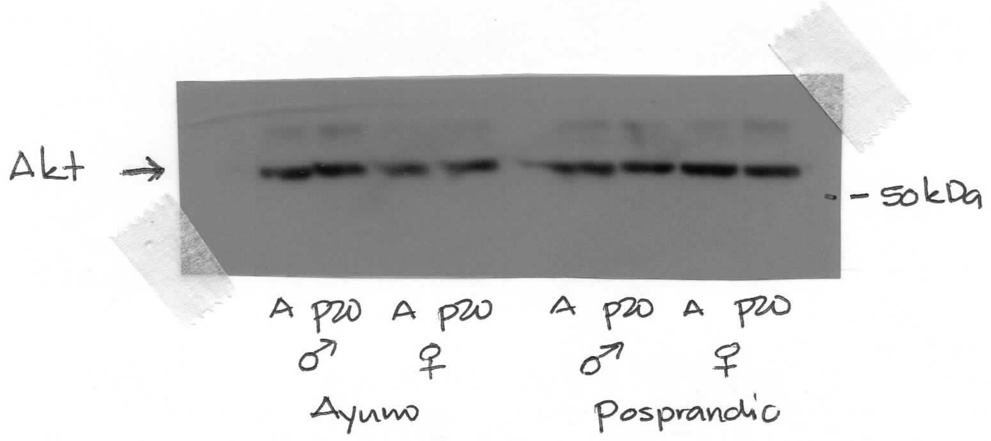

Tejido Adiposo Peripancreático
16 Mayo 2018
25 Mayo 2018

p-IRS1 (S1101) →

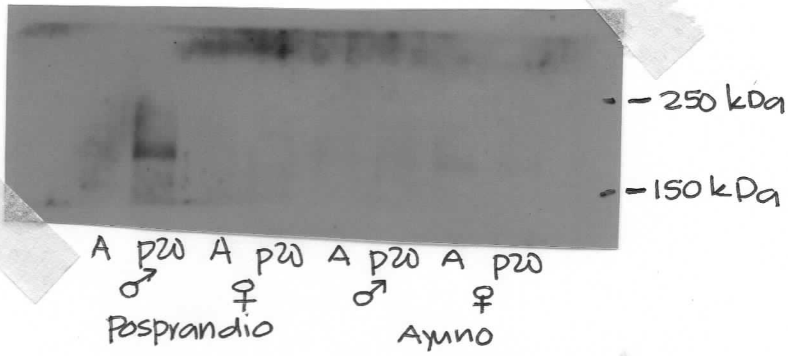

— 250 kDa
— 150 kDa

A p20 A p20 A p20 A p20
♂ ♀ ♂ ♀
Posprandio Ayuno

IRS1 →

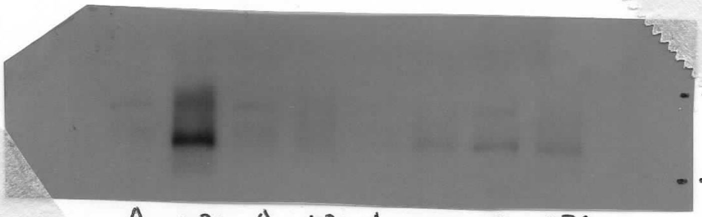

— 250 kDa
— 150 kDa

A p20 A p20 A p20 A p20
♂ ♀ ♂ ♀
Posprandio Ayuno

p-Akt (S473) →

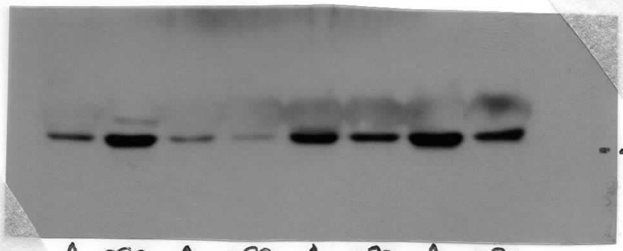

— 50 kDa

A p20 A p20 A p20 A p20
♂ ♀ ♂ ♀
ayuno posprandio

actina2 →

actina1 →

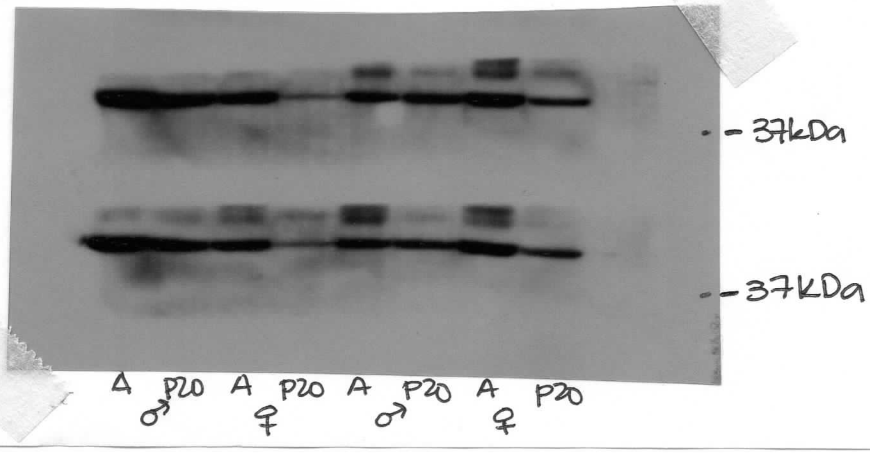

— 37 kDa
— 37 kDa

A p20 A p20 A p20 A p20
♂ ♀ ♂ ♀

Tejido Adiposo Peripancreático
13 Junio 2018

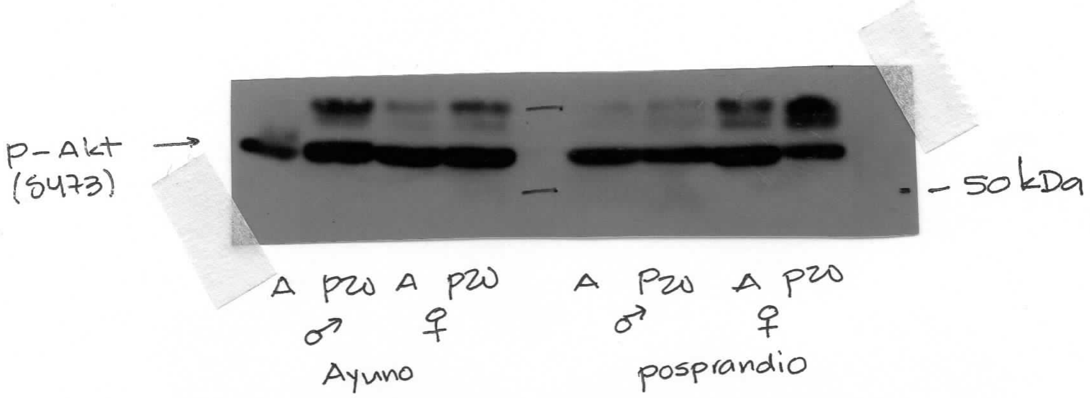

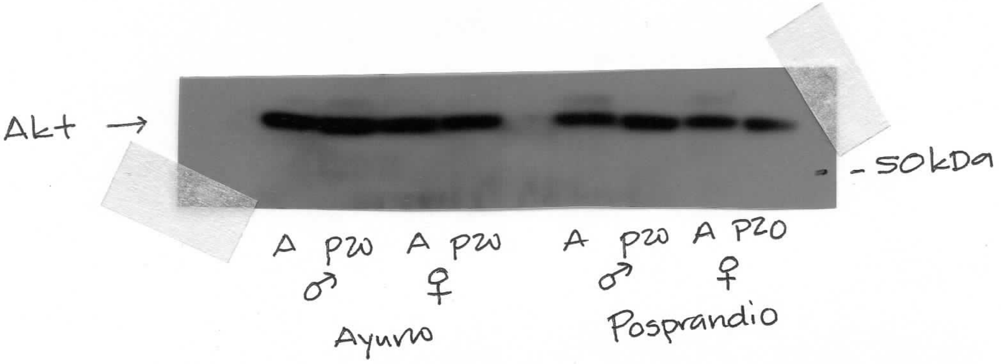

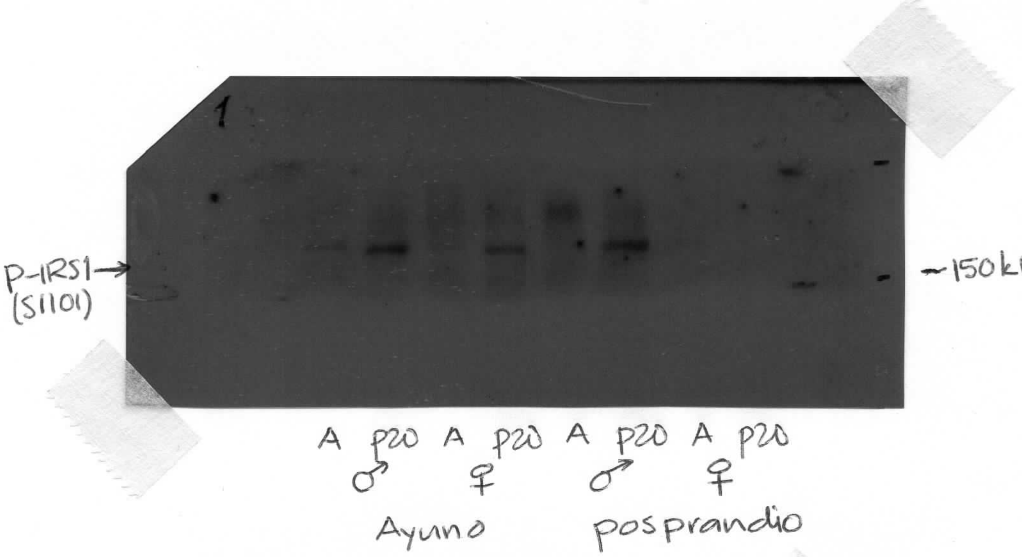

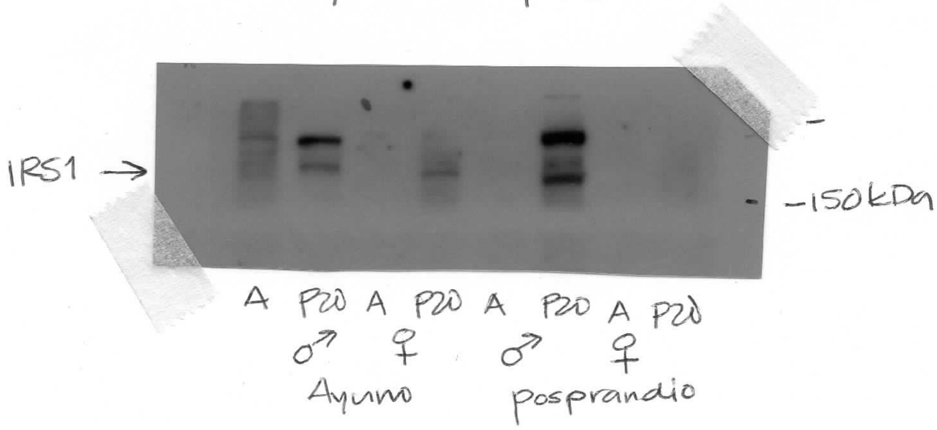

Tejido Adiposo peripancreático.
13 Junio 2018

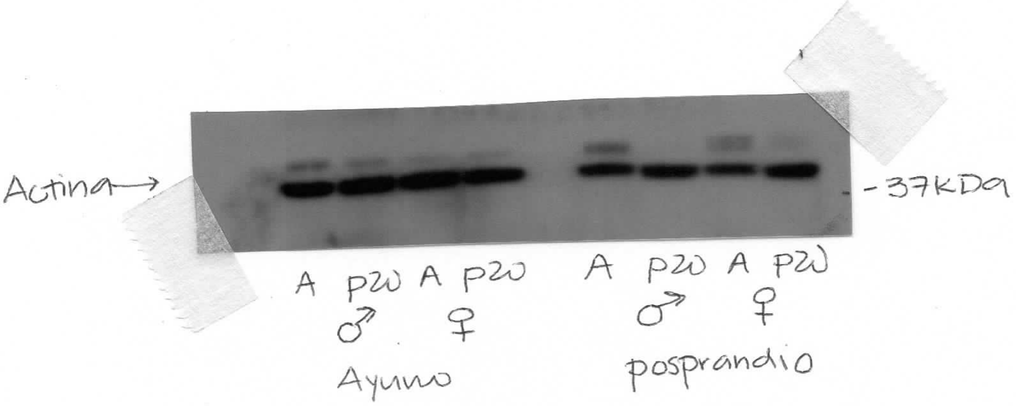

Músculo Gastrocnemio
Nov / Oct 2018

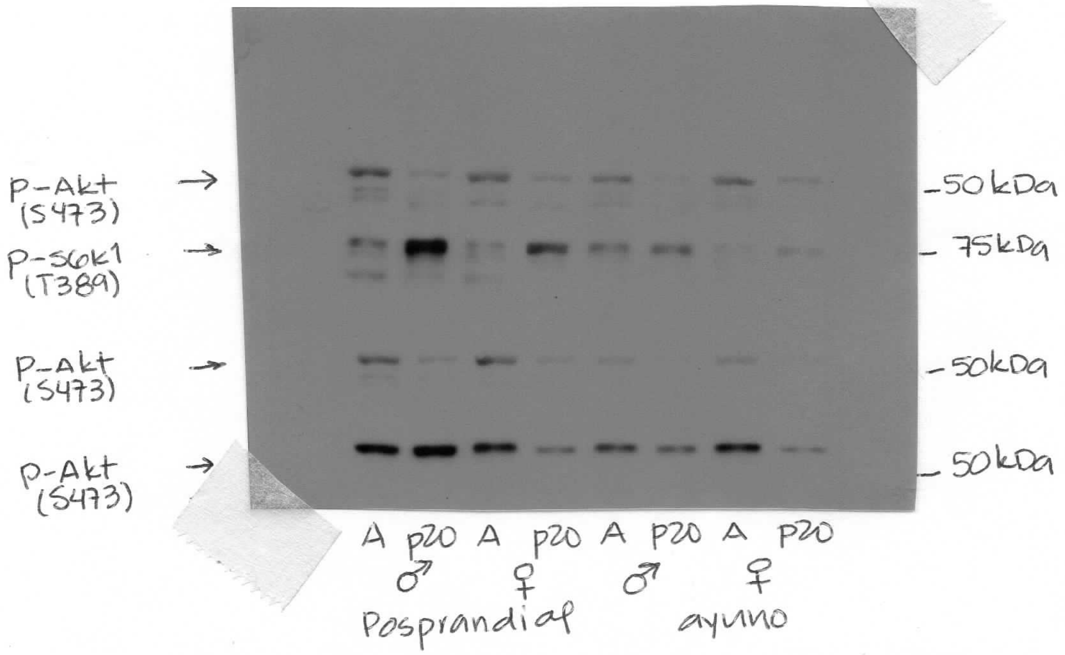

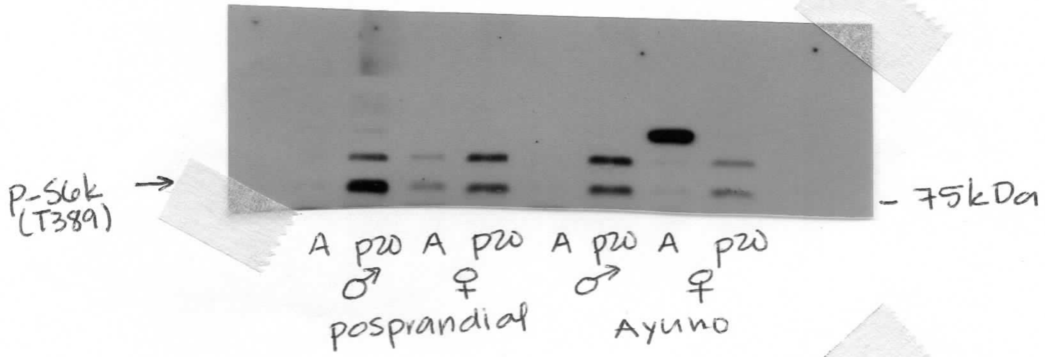

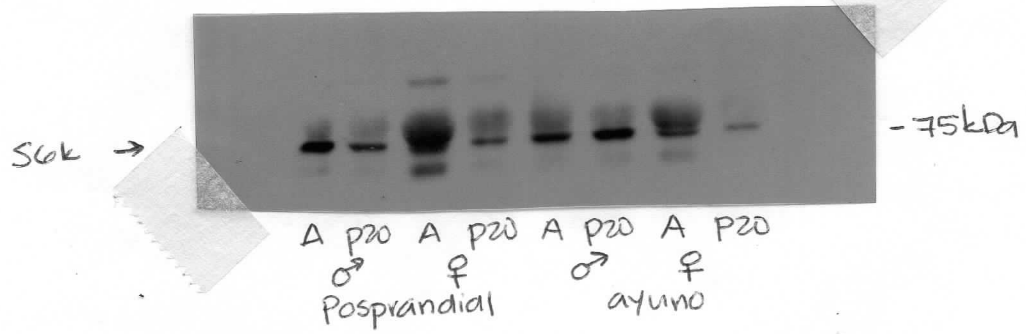

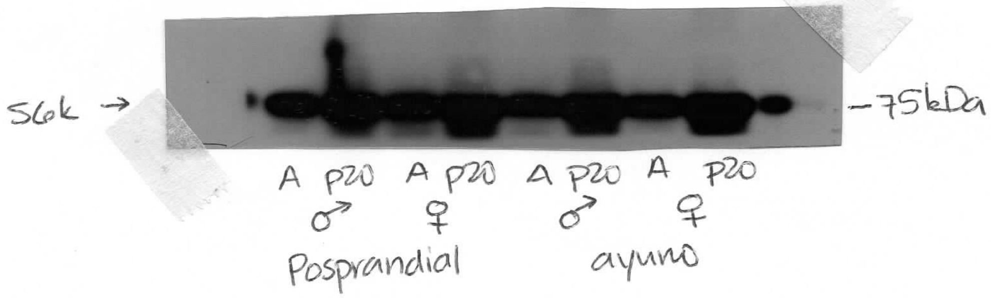

Músculo Gastrocnemio
Nov / Oct 2018

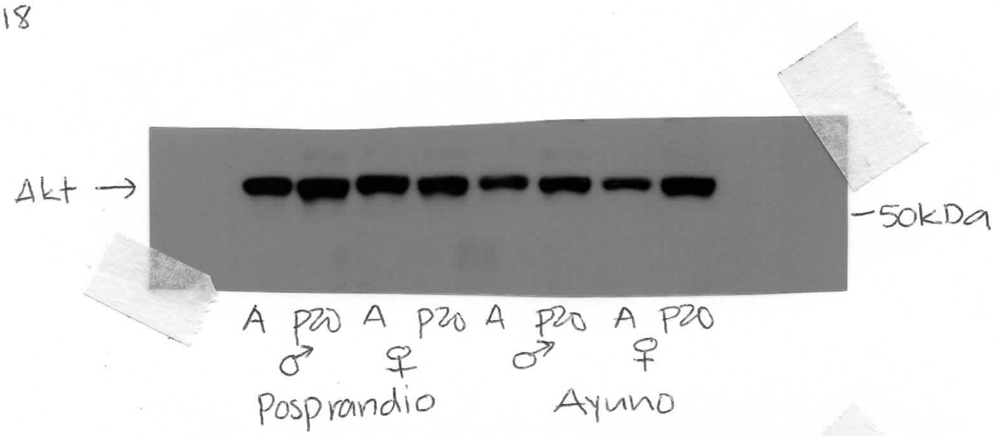

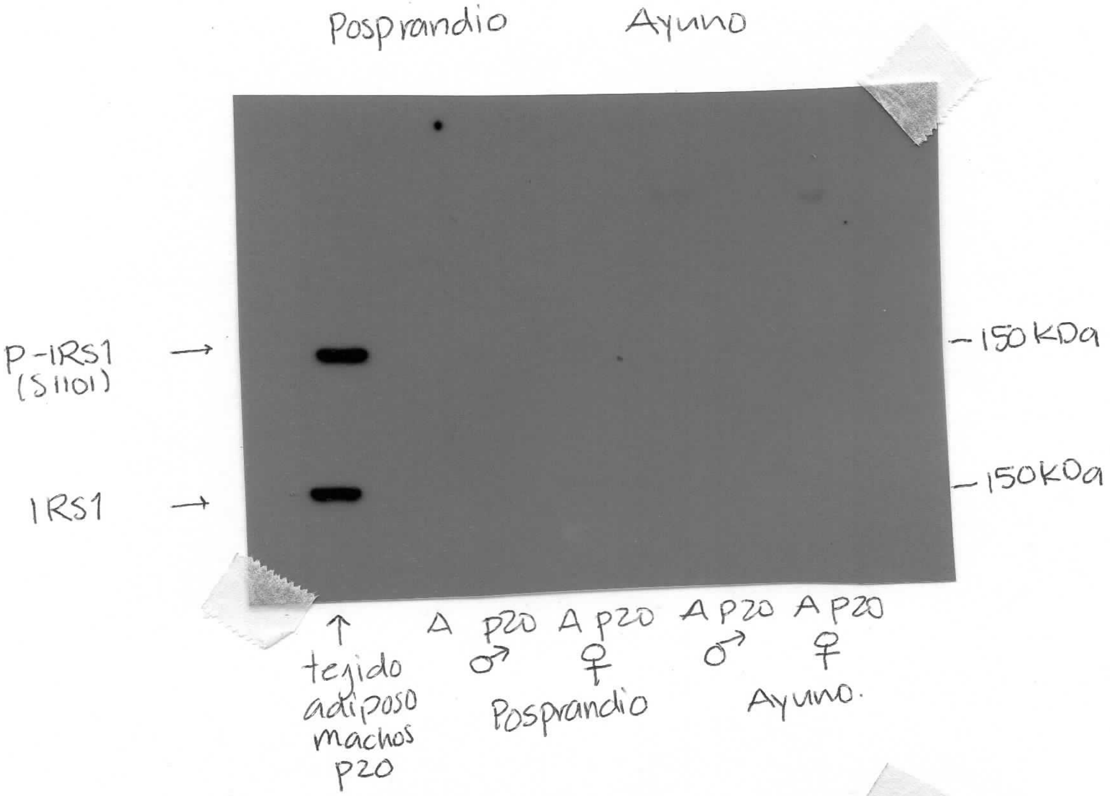

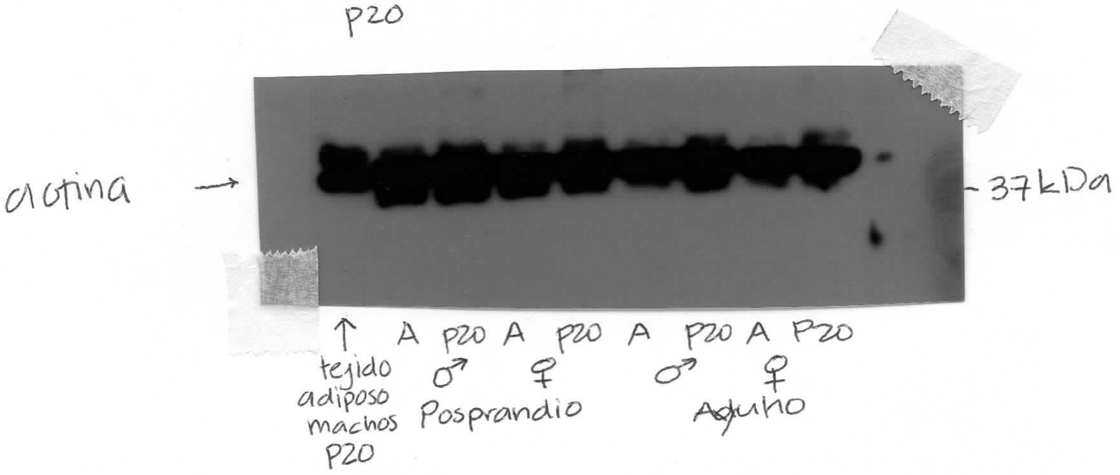

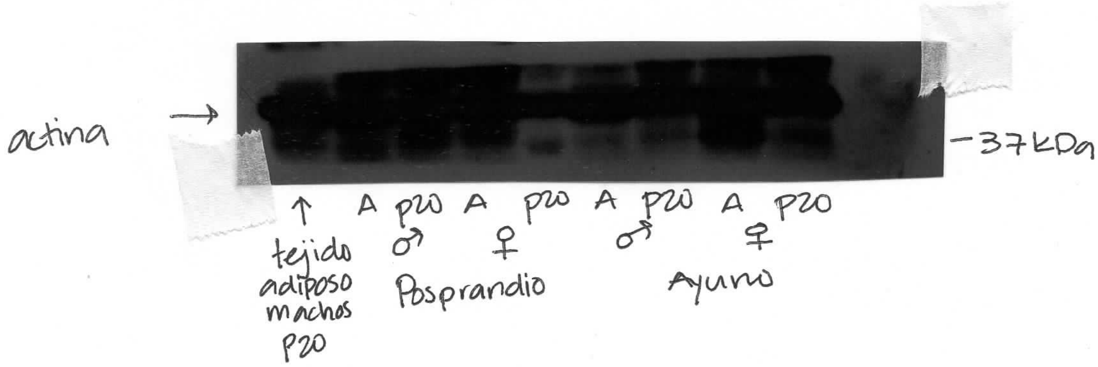

Músculo Gastrocnemio

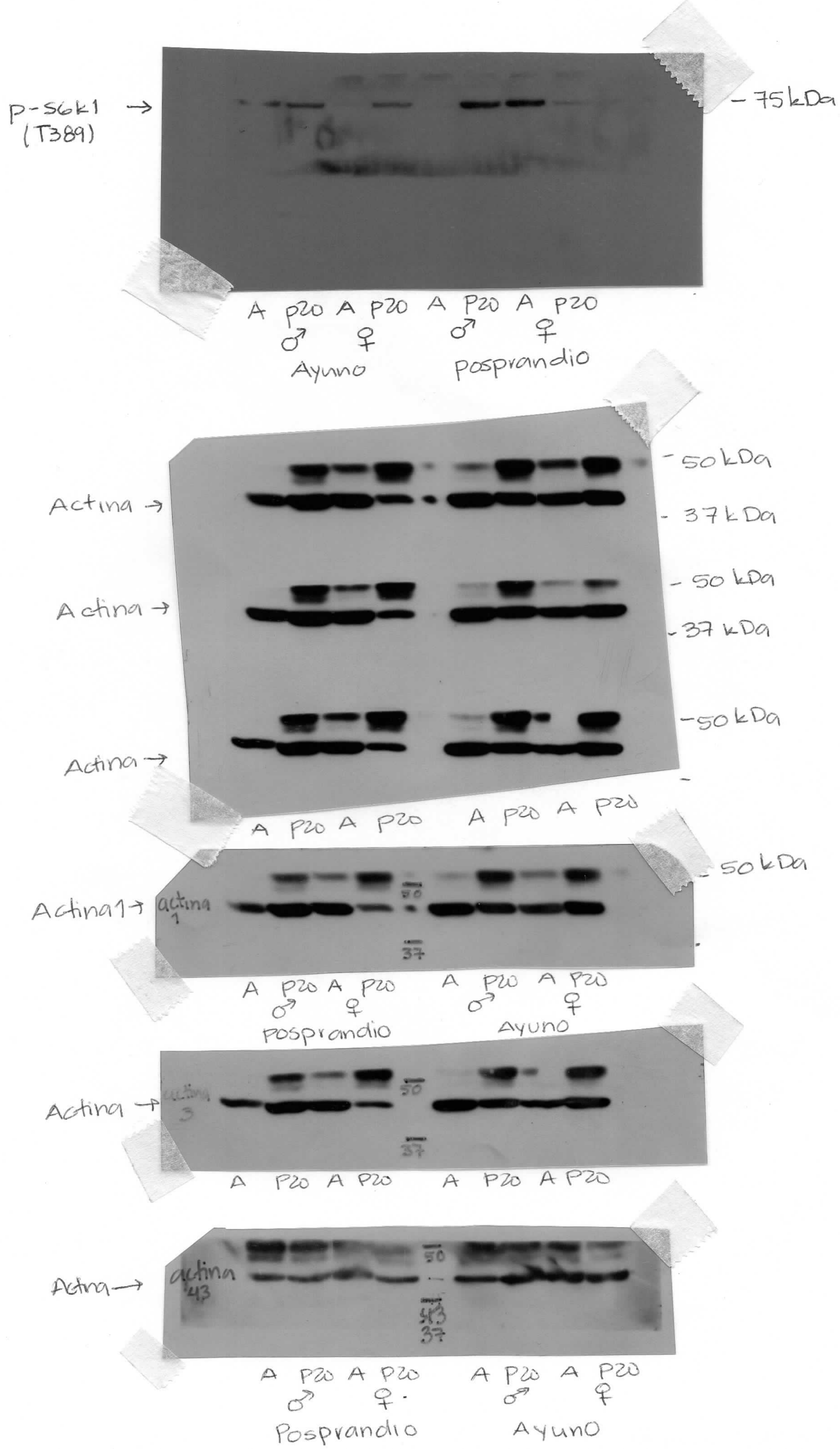

Musculo gastrocnemio

Oct 2018

Actina →

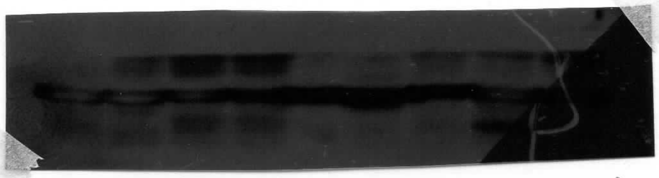

A P20 A P20 A P20 A P20 P20

♂ ♀ ♂ ♀

Posprandio ayuno
